# Estrogen Suppresses Cytokines Release in cc4821 *Neisseria meningitidis* Infection *via* TLR4 and ERβ-p38-MAPK Pathway

**DOI:** 10.3389/fmicb.2022.834091

**Published:** 2022-03-29

**Authors:** Pengbo Guo, Juan Xu, Hao Liang, Li Xu, Wanying Gao, Ziman Chen, Yuan Gao, Maojun Zhang, Guangfu Yu, Zhujun Shao

**Affiliations:** ^1^State Key Laboratory for Infectious Disease Prevention and Control, National Institute for Communicable Disease Control and Prevention, Chinese Center for Disease Control and Prevention, Beijing, China; ^2^Department of Pathogen Biology, School of Basic Medicine, Shandong First Medical University and Shandong Academy of Medical Sciences, Jinan, China; ^3^Department of Microbiology, School of Public Health, Cheeloo College of Medicine, Shandong University, Jinan, China; ^4^Collaborative Innovation Center for Diagnosis and Treatment of Infectious Diseases, Hangzhou, China

**Keywords:** estrogen, *N. meningitidis*, p38-MAPK, TLR4, ERβ

## Abstract

Estrogen has long been known to possess immune-modulatory effects in diseases, and multiple pathological conditions show great sex disparities. However, the impact of estrogen in *Neisseria meningitidis* infection has not been determined. The present study aimed to investigate the role of estrogen in *N. meningitidis* infection and the molecular mechanism. We selected 35 *N. meningitidis* isolates representing different clonal complexes (cc), serogroups, and isolation sources to infect the HBMEC cell line. Results showed that the expression of estrogen receptor (ER) β in *N. meningitidis*-infected cells was downregulated compared with that in normal cells. The expression of ERβ induced by invasive isolates was lower than that in carriers. Serogroup C isolates induced the lowest expression of ERβ compared with serogroup A and B isolates. We used four cc4821 *N. meningitidis* isolates to infect two kinds of host cells (human brain microvascular endothelial cells and meningeal epithelial cells). The results showed that 17 β-estradiol (E2) could inhibit the release of inflammatory factors interleukin (IL)-6, IL-8, and tumor necrosis factor-α after *N. meningitidis* infection *via* TLR4. E2 could inhibit the activation of the p38-MAPK signal pathway induced by *N. meningitidis* infection through binding to ERβ, and significantly inhibit the release of inflammatory factors in *N. meningitidis*-infected host cells. This study demonstrated that estrogen plays a protective role in *N. meningitidis* infection. ERβ is potentially associated with the release of inflammatory cytokines in *N. meningitidis* infection, which sheds light on a possible therapeutic strategy for the treatment of invasive diseases caused by *N. meningitidis*.

## Introduction

As a sex hormone that induces growth and development, estrogen is expressed in both men and women, and participates in regulating a variety of physiological and pathological processes, including adenocarcinoma, pathogenic microbial infectious diseases, neurodegeneration, and inflammation. The cellular effect of estrogen is mediated by the estrogen receptor (ER). The two dominant receptors are ERα and ERβ (Pazos et al., 2012; Rouphael and Stephens, 2012; Almey et al., 2015; Santos et al., 2017; Patel et al., 2018).

ERα and ERβ have both synergistic and antagonistic effects. They work together to maintain homeostasis (Rouphael and Stephens, 2012; Roberts et al., 2013; Pizza and Rappuoli, 2015; Patel et al., 2018). When estrogen binds to ER, it initiates transcriptional regulation. Activated ER regulates the nuclear factor kappa B (NF-κB) signaling pathway, upregulates transcription factor activating protein-1, and further activates many downstream signal pathways, such as the AMP-activated protein kinase (AMPK) signaling pathway and mitogen activated protein kinase (MAPK) signaling pathway (Imamov et al., 2005). Upregulated transcription factor activating protein-1 induces the secretion of inflammatory factors and increases the expression of vascular endothelial growth factor (Patel et al., 2018). In addition, the ERβ promoter region contains many CpG islands, which are the binding targets of different methylation patterns (Chisamore et al., 2012; Gianchecchi et al., 2015; Ali et al., 2017; Chakhtoura et al., 2017; Zouheir et al., 2019).

*Neisseria meningitidis* (*N. meningitidis*) is a bacteria that colonizes the human nasopharyngeal mucosa, with human as the only host, and is also the main cause of invasive meningococcal disease (IMD). Therefore, *N. meningitidis* is considered to be an opportunistic pathogen. *N. meningitidis* can be divided into different sequence types (STs) based on the sequences of its seven housekeeping genes. STs with four or more identical loci can be classified into the same clonal complex (cc) (Shao et al., 2006). Previous molecular epidemiological studies have found that isolates of ccs, such as cc5, cc11, cc32, cc41/44, and cc4821, are more likely to cause IMD; therefore, these ccs are also called hyper-invasive ccs. Cc4821 isolates were one of the most important hyper-invasive isolates in China. The interaction between *N. meningitidis* and host cells is critical in the pathogenicity process of *N. meningitidis* (Shao et al., 2006; Guo et al., 2019).

Previous studies have found that estrogen is associated with the occurrence, development, and prognosis of some infectious diseases, autoimmune diseases, and malignant tumors (Imamov et al., 2005; Pazos et al., 2012; Almey et al., 2015; Kovats, 2015; Santos et al., 2017). Investigate of the potential role played by estrogen in *N. meningitidis* infection and its underlying mechanism will not only clarify the interaction between *N. meningitidis* and host cells at the molecular level, but also shed light on the clinical treatment of critical sequelae caused by *N. meningitidis* infection.

Here, we investigated the role and the molecular mechanism of estrogen in *N. meningitidis* infection. We evaluated the expression of ERβ in *N. meningitidis*-infected cells and the relationship between its expression and *N. meningitidis* characteristics. Our results revealed the regulatory role of estrogen in *N. meningitidis* infection and provided the clues for new therapeutic measures to treat severe IMDs.

## Materials and Methods

### Studied Isolates and Growth Conditions

Thirty-five *N. meningitidis* isolates were cultured on a Columbia 5% sheep blood agar plates (BAPs) (Thermo Fisher Oxoid, Beijing, China) and incubated at 37°C with 5% CO_2_ for 24 h. Information related to the 35 isolates used in this study is provided in Table 1. All experiments were performed in a safety cabinet.

**TABLE 1:** ∣ *Neisseria meningitidis* isolates used in this study.

Isolates ID	ST	Cc	Serogroup	Source
320503	4821	4821	C	Invasive isolate
340542	4821	4821	C	Invasive isolate
100603	4821	4821	C	Invasive isolate
341215	4821	4821	B	Invasive isolate
321114	3200	4821	B	Invasive isolate
370601	3200	4821	C	Invasive isolate
431210	4821	4821	B	Invasive isolate
330505	4896	4821	C	Invasive isolate
340552	4897	4821	B	Carried isolate
100572	5610	4821	C	Carried isolate
100514	4832	4821	C	Carried isolate
130803	6928	4821	C	Carried isolate
421102	3200	4821	B	Carried isolate
360624	5473	4821	C	Carried isolate
420703	12311	4821	B	Carried isolate
320501	4820	4821	C	Invasive isolate
420718	11920	4821	C	Invasive isolate
440902	4821	4821	B	Invasive isolate
440529	7	5	A	Invasive isolate
130508	7	5	A	Invasive isolate
310501	7	5	A	Invasive isolate
510612	7	5	A	Invasive isolate
100806	2859	5	A	Invasive isolate
651801	7	5	A	Invasive isolate
150720	2146	198	Cnl	Carried isolate
130817	2146	198	Cnl	Carried isolate
340809	2146	198	Cnl	Invasive isolate
211002	2146	198	Cnl	Carried isolate
341403	4821	4821	C	Invasive isolate
341215	4821	4821	B	Invasive isolate
LNT3	7	5	A	Invasive isolate
440530	7	5	A	Invasive isolate
421401	12316	4821	B	Invasive isolate
421007	4821	4821	B	Carried isolate
321102	4821	4821	C	Invasive isolate

### Cell Culture, E2 Treatment, and shRNA Transfection

Human brain microvascular endothelial cells (HBMECs) and meningeal epithelial cells (MECs) were purchased from ATCC (ATCC-CRL-2922, Manassas, VA, United States) (Shanghai, China; catalog no. AT3190; Shanghai, China; catalog no. AT4122). HBMECs were cultured in Dulbecco’s modified Eagle medium (DMEM) (Gibco by Invitrogen, Carlsbad, CA, United States) supplemented with 10% fetal bovine serum (FBS) (catalog no. FS201-02; Transgen Biotech, Beijing, China) and 1% penicillin-streptomycin solution (final concentrations: Penicillin, 100 units/ml; streptomycin, 100 μg/ml) (catalog no. 10378016; Thermo Fisher Scientific, Shanghai, China). MECs were cultured in Roswell Park Memorial Institute (RPMI) 1640 Medium supplemented with the same concentration of FBS and penicillin-streptomycin solution. The growth conditions for both cell lines consisted of a humid atmosphere with 5% CO_2_ at 37°C.

Both cells were seeded in 6-well plates and incubated for 20 h before treatment with 50 nM of estradiol-17β (E2) (E2758, Sigma, St Louis, MO, United States) for another 24 h before being harvested for subsequent experiments.

The short hairpin RNA (shRNA) targeting *TLR4* (Toll like receptor 4; Shanghai Genechem Co., Ltd., Shanghai, China) was transfected into the HBMECs and MECs to block TLR4 expression, and random sequences were used as non-specific control (Si-NC). The efficiency of downregulation of TLR4 was verified using western blot. All of the transfections were performed using Lipofectamine 3000 (Invitrogen, Thermo Fisher Scientific, Shanghai, China) according to the manufacturer’s instructions.

### Infection Experiments

Human brain microvascular endothelial cells and MECs were seeded into 6-well cell culture plates at a density of 2 × 10^5^ cells/well. The day before infection, the cell lines were starved by culturing them in opti-MEM I Reduced-Serum Medium (31985070, Thermo Fisher Scientific). On the next day, a suspension of the overnight bacterial culture in opti-MEM I was adjusted to an optical density of 0.200 at 600 nm. After 1 × 10^5^ dilution, 100 μL of the bacteria suspension was streaked on a BAP and incubated at 37°C with 5% CO_2_ for 24 h to calculate the initial number of bacteria infecting the host cells. Bacterial suspension (2 mL) was added into each of the eleven wells and further incubated at 37°C with 5% CO_2_. One well containing non-infected cells was included in each experiment as a control (CTR). After 4 h of infection, unbound bacteria were washed away using sterile phosphate-buffered saline (PBS) three times.

### Inhibitor Treatment

For ERβ inhibitor treatment, HBMECs and MECs were pretreated with ERβ inhibitor PHTPP (1 μM) (#ab145148, Abcam, Cambridge, MA, United States) for 30 min before E2 treatment for 24 h.

For p38-MAPK signaling pathway inhibitor treatment, HBMECs and MECs were pretreated with p38 inhibitor SB203580 (20 μM) (#ab120162, Abcam) before E2 treatment for 24 h.

### Western Blotting for Signaling Pathway Screening

Human brain microvascular endothelial cells and MECs were collected after 48 h of infection. The total protein was extracted from both cell types and lysed in Radioimmunoprecipitation assay (RIPA) lysis buffer (Beyotime, Hayman, China). The cell extract was centrifuged at 12,500 × *g* at 4°C for 25 min. Thereafter, the total protein (60 μg) was fractionated on 12% SDS-PAGE gels and transferred to polyvinylidene difluoride membranes for western blotting. Western blotting was performed by using a specific Phospho-MAPK Family antibody sampler kit (#9910) from Cell Signaling Technology (Danvers, MA, United States) to detect MAPK signaling pathway-related proteins phospho (p)-ERK (extracellular regulated kinase), p-JNK (JUN N-terminal kinase) and p-p38. Primary antibodies against the TNF-α (#6945) was also purchased from Cell Signaling Technology Inc. The primary antibodies against ERβ (#ab3576) and TLR4 (#ab13556) were purchased from Abcam company. β-actin was detected as an internal control using a mouse anti-β-actin monoclonal antibody (#HC201-02, TransGen Biotech, Beijing, China). The secondary antibodies against mouse IgG (#7076) and rabbit IgG (#7074) were purchased from Cell Signaling Technology Inc.

### Enzyme-Linked Immunosorbent Assay of Cytokines

After E2 treatment and *N. meningitidis* isolate infection, both cell types were further cultured for 48 h. Cytokines were detected using ELISA. The interleukin-6 (IL-6) level was detected using Human IL-6 ELISA Set (#555220, BD Bioscience, San Diego, CA, United States). The release of interleukin-8 (IL-8) was detected using a Human IL-8 ELISA Kit II (#550999, BD Bioscience). The TNF-α level was detected using a Human TNF ELISA Kit II (#550610, BD Bioscience). The cytokines mentioned above were all detected according to the manufacturer’s instructions and run in triplicate. The above-mentioned cytokine levels released by non-infected HBMECs and MECs were measured as controls.

### Statistical Analyses

All analyses were performed using SPSS version 20.0 software (IBM Corp., Armonk, NY, United States) and GraphPad Prism 5 (GraphPad, La Jolla, CA, United States). Differences between means were compared and analyzed using a two-way analysis of variance (ANOVA) assay. A two-sided *p*-value of <0.05 was considered statistically significant.

## Results

### ERβ Downregulated in *Neisseria meningitidis* Infection

**FIGURE 1:**
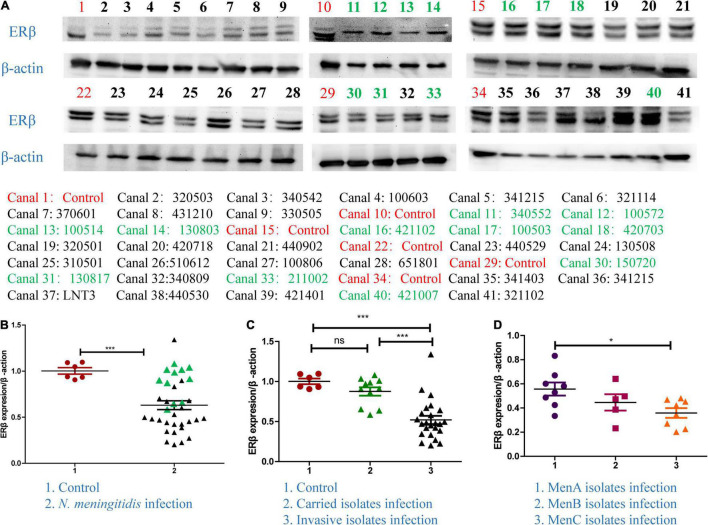
The expression of ERβ in *Neisseria meningitidis*-infected cells. **(A,B)** Thirty-five *N. meningitidis* isolates were collected from China CDC, and host cells were infected with the *N. meningitidis* isolates. The proteins of the 35 infected-cells were extracted, and the cellular proteins of uninfected *N. meningitidis* as the control group (CTR) were extracted. Isolates ID with black color represented invasive isolates. Isolates ID with green color represented invasive isolates. Western blotting assays were used to detect the levels of ERβ in host cells after infection with *N. meningitidis*. ***P < 0.001. **(C)** Graph-pad Prism software further analyzed the levels of ERβ in the control group, the carrier isolates infection group, and invasive isolates infection group. Ns, not-significant, ***P < 0.001. **(D)** The above software was used to analyze the changes of ERβ levels in host cells after infection with serogroup A, B, and C invasive isolates. *P < 0.05.

In order to identify whether E2 participated in *N. meningitidis* infection, we determined the protein level of ERβ in *N. meningitidis* isolate-infected HBMEC cells and non-infected cells. The western blotting data showed that the ERβ level was significantly downregulated in infected cells compared with that in normal cells from a cohort of 35 different *N. meningitidis* isolates (^***^P < 0.001) (Figures 1A,B). Data analysis showed that invasive isolates decreased the level of ERβ to a greater extent than the carried isolates (column 2 vs. 3, ^***^P < 0.001) (Figure 1C), while the level of ERβ showed no significant difference after carried isolates infection compared with the CTR (column 1 vs. 2, *P* > 0.05) (Figure 1C). Comparing the ERβ level in response to MenA, MenB, and MenC invasive isolates infection showed that infection with MenC invasive isolates resulted in the lowest ERβ expression (*P < 0.05) (Figure 1D).

### The Effect of E2 on Inflammatory Cytokines Release After *Neisseria meningitidis* Infection

**FIGURE 2:**
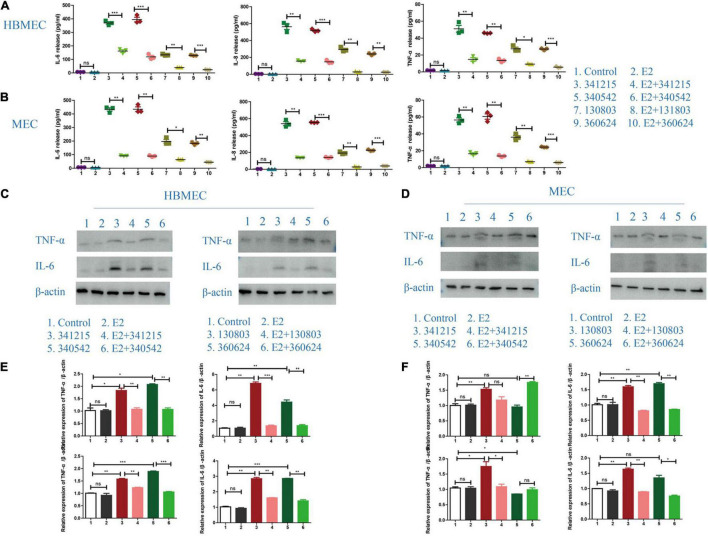
Inflammatory cytokines release in *Neisseria meningitidis*-infected cells after E2 treatment. **(A,B)** HBMECs **(A)** and MECs **(B)** were treated with *N. meningitidis* infection and *N. meningitidis* infection after E2 pretreatment. Cells without *N. meningitidis* infection and the cells stimulated by E2 only were used as controls. ELISA assays were used to detect the expression of inflammatory factors IL-6, IL-8, and TNF-α in the culture supernatant. **(C–F)** HBMECs **(C)** and MECs **(D)** were treated as above. Western blotting was used to detect the levels of TNF-α and IL-6, with β-actin used as a loading control. The levels of the above molecules in both cells was analyzed by Image-J software, and the gray analysis value was analyzed statistically using Graph-pad Prism software. NS (not significant) is *P* > 0.05, *P < 0.05, **P < 0.01, ***P < 0.001.

To define the role of E2 in inflammatory factors release after *N. meningitidis* infection, we treated the HBMECs and MECs with E2 before *N. meningitidis* isolate infection and detected the expression of inflammatory factors. The ELISA results showed that E2 treatment did not influence the production of inflammatory cytokines in uninfected cells (*P* > 0.05, lane 1 vs. 2, Figures 2A,B). Compared with *N. meningitidis* isolate-infected cells (lanes 3, 5, 7, and 9), expression of IL-6, IL-8, and TNF-α were significantly decreased in E2-pretreated-infected cells (lanes 4, 6, 8, and 10, *P < 0.05, ^**^P < 0.01, ^***^P < 0.001, Figures 2A,B), which was further confirmed using western blotting (Figures 2C–F).

### The Effect of E2 on p38-MAPK Pathway in *Neisseria meningitidis*-Infected Cells

**FIGURE 3:**
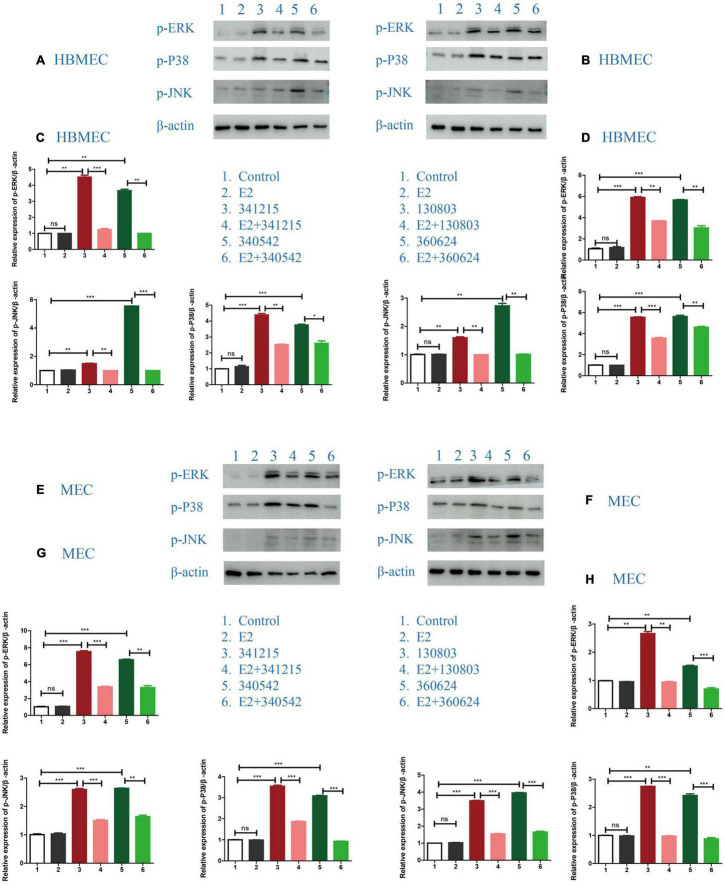
The effect of E2 on the MAPK signaling pathway in *Neisseria meningitidis*-infected cells. **(A,B)** HBMECs were treated with *N. meningitidis* isolates 341215 and 340542 **(A)**, 130803 and 360624 **(B)**. The levels of phosphorylated ERK, JNK, and P38, as MAPK downstream molecules, in host cells were detected using western blotting, with β-actin used as the control. **(C,D)** The changes of protein levels in HBMECs were analyzed using Image-J software, and the gray values were statistically analyzed using GraphPad Prism software. **(E–H)** The levels of downstream molecules of the MAPK pathway were detected by western blotting in MECs according to the above treatment scheme. *P < 0.05, **P < 0.01, ***P < 0.001.

Next, we attempted to define the signaling pathway through which E2 could confer anti-inflammatory effects on the cells. The results for both the hyper-invasive (ID: 341215 and 340542) and hypo-invasive (ID: 130803 and 360624) *N. meningitidis* isolates infection demonstrated that E2 effectively suppressed the activated-MAPK pathway, as evaluated by its downstream signaling molecules, including p-ERK, p-JNK and p-P38, in both HBMECs and MECs (Figure 3).

### The Role of p38-MAPK Pathway in the Effect of E2 in *Neisseria meningitidis*

To test whether E2-mediated reverted the inflammatory behavior of *N. meningitidis*-infected cells through MAPK pathway, we pretreated HBMECs and MECs with the p38 inhibitor SB203580 before treatment with E2 and *N. meningitidis*. The cells without inhibitor pretreatment were used as the control group. The inhibitory effect was verified by western blotting.

**FIGURE 4:**
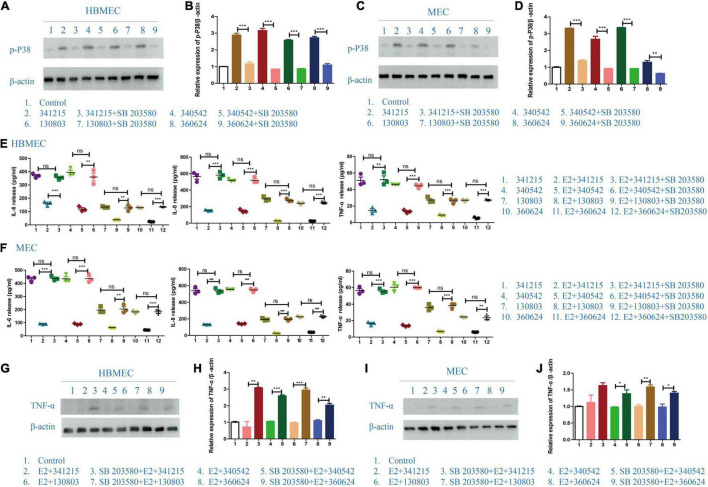
The effect of p38-MAPK signaling pathway on the role of E2 in *Neisseria meningitidis*-infected cells. **(A–D)** P38-MAPK signal pathway inhibitor, SB203580, was used in *N. meningitidis*-infected HBMECs **(A)** and MECs **(C)**. Proteins were extracted for western blotting analysis. The activation of p-p38 was detected and β-actin was used as the internal control. The changes in protein levels in HBMECs **(B)** and MECs **(D)** were analyzed using Image-J software and the gray values were analyzed statistically using GraphPad Prism software. **(E,F)** HBMECs **(E)** and MECs **(F)** were treated with the following three treatments: *N. meningitidis* infection, E2-*N. meningitidis* co-stimulation, SB203580-E2-*N. meningitidis* co-stimulation, and the cell supernatant was collected. ELISA assays were used to detect the levels of IL-6, IL-8, and TNF-α in the supernatant. **(G–J)** HBMECs **(G)** and MECs **(I)** were divided into three experimental groups as shown. Extracted proteins were subjected to western blotting. The level of TNF-α was detected and β-actin was used as an internal control. The changes of protein levels in both cells **(H,J)** were analyzed as above. *P < 0.05, **P < 0.01, ***P < 0.001.

Western blotting data showed that SB203580 could effectively suppress activated-p-p38 (^**^P < 0.01, ^***^P < 0.001, Figures 4A–D). ELISA data showed that when p38 was inhibited by SB203580, E2 lost its protective effect in *N. meningitidis* infection (lane 2 vs. 3, lane 5 vs. 6, lane 8 vs. 9, lane 11 vs. 12, *P < 0.01, ^***^P < 0.001, Figures 4E,F). The ELISA result was confirmed by western blotting, which suggested that TNF-α expression was upregulated when HBMECs and MECs were pretreated with SB203580 (lane 2 vs. 3, lane 4 vs. 5, lane 6 vs. 7, lane 8 vs. 9, *P < 0.05, ^**^P < 0.01, ^***^P < 0.001, Figures 4G–J).

### The Effect of TLR4 in E2 Protection in *Neisseria meningitidis* Infection

**FIGURE 5:**
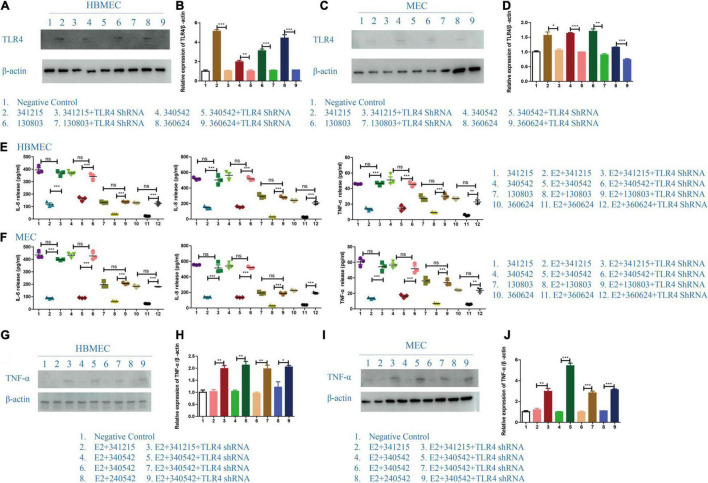
The effect of TLR4 on the role of E2 in *Neisseria meningitidis*-infected cells. **(A–D)** *TLR4* shRNA was transfected in to HBMECs **(A)** and MECs **(C)** infected with *N. meningitidis*. The cells infected with *N. meningitidis* without *TLR4* shRNA transfection and the cells transfected with negative control were used as controls. Proteins were extracted and the level of TLR4 was detected using western blotting. β-actin was used as the internal reference. The changes in protein levels in HBMECs **(B)** and MECs **(D)** were analyzed using Image-J software, and the gray values were analyzed statistically using GraphPad Prism software. **(E,F)** A *TLR4* shRNA was transfected into E2-*N. meningitidis* co-stimulated HBMECs **(E)** and MECs **(F)**. The E2-*N. meningitidis* co-stimulation experimental group without shRNA transfection and the experimental group only infected with *N. meningitidis* were used as controls. The supernatants of the cells were collected for ELISA assays, and the levels of IL-6, IL-8, and TNF-α in the supernatants were detected. **(G–J)** HBMECs **(G)** and MECs **(I)** were treated as shown. The expression of TNF-α was detected using western blotting. β-actin was the internal reference. The changes in protein levels in both cells **(H,J)** were analyzed as above. *P < 0.05, **P < 0.01, ***P < 0.001.

To test the hypothesis that TLR4 is the key TLR in E2’s protective effect against *N. meningitidis*, we examined the production of IL-6, IL-8, and TNF-α by HBMECs and MECs following treatment with a *TLR4* shRNA. TLR4 inhibition was verified by western blotting (Figures 5A–D; *P < 0.05, ^**^P < 0.01, ^***^P < 0.001).

The ELISA results showed that compared with *N. meningitidis* isolate-infected cells without *TLR4* shRNA transfection (lanes 2, 5, 8, and 11), the levels of IL-6, IL-8, and TNF-α were significantly increased in *TLR4* shRNA transfection cells (lanes 3, 6, 9, and 12, ^**^P < 0.01, ^***^P < 0.001, Figures 5E,F), which was further confirmed using western blotting (lane 2 vs. 3, lane 4 vs. 5, lane 6 vs. 7, lane 8 vs. 9, *P < 0.05, ^**^P < 0.01, ^***^P < 0.001, Figures 5G–J).

These experimental results showed that after the effective inhibition of TLR4, estrogen cannot downregulate the inflammatory factors of the host cells in the *N. meningitidis*-infected host cells. These results further suggested that estrogen plays a role in *N. meningitidis* infection by TLR4 participating.

### The Role of ERβ in *Neisseria meningitidis* Infection

**FIGURE 6:**
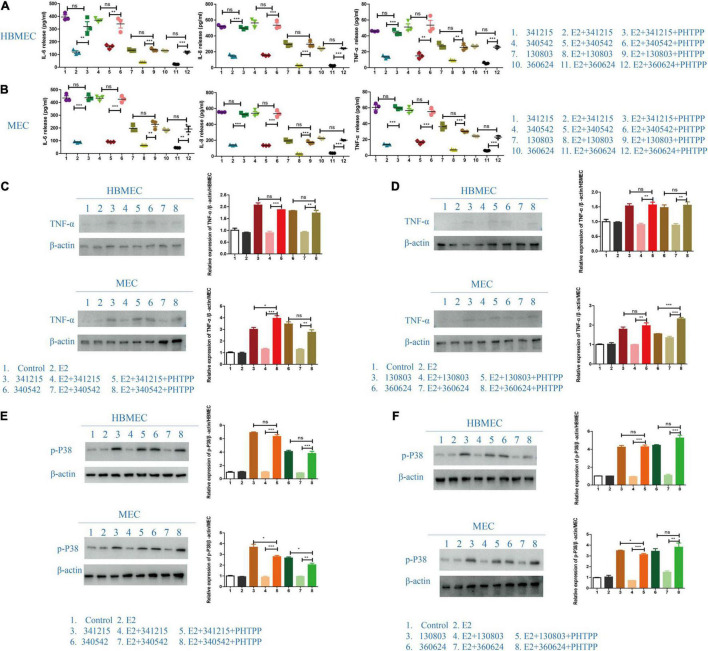
The effect of ERβ on the role of E2 in *Neisseria meningitidis*-infected cells. **(A,B)** HBMECs **(A)** and MECs **(B)** were divided into three experimental groups: *N. meningitidis* infection group, E2-*N. meningitidis* co-stimulation group, and PHTPP-E2-*N. meningitidis* co-stimulation group. The supernatant was extracted and the levels of IL-6, IL-8, and TNF-α in the supernatant was detected using ELISA assays. **(C–F)** HBMECs and MECs were treated as shown. Cells (CTR) without any treatment and the cells treated with E2 were used as controls, and proteins were extracted for western blotting. The levels of TNF-α and p-p38 were detected and β-actin was used as the internal control. The changes in protein levels were analyzed as above. *P < 0.05, **P < 0.01, ***P < 0.001.

To further verify the role of ERβ in *N. meningitidis* infection, we aimed to reverse the effect of ERβ on *N. meningitidis* infection by inhibiting the activity of ERβ. PHTPP, a specific inhibitor of ERβ, was used to block the activity of ERβ. ELISA and western blotting tests were used to detect the release of inflammatory factors from host cells. The results showed that the levels of IL-6, IL-8, and TNF-α in the PHTPP-estrogen-*N. meningitidis* co-stimulation group were significantly higher than those in the estrogen-*N. meningitidis* co-stimulation group (^**^P < 0.01, ^***^P < 0.001, sample 2 vs. 3, 5 vs. 6, 8 vs. 9, 11 vs. 12, Figures 6A,B). Compared with the experimental group only infected with *N. meningitidis*, there was no difference in the release of these factors between the PHTPP-estrogen-*N. meningitidis* experimental group and its host cells (*P* > 0.05, sample 1 vs. 3, 4 vs. 6, 7 vs. 9, 10 vs. 12, Figures 6A,B). The results of western blotting were consistent with those of the ELISA experiment (^**^P < 0.01, ^***^P < 0.001, sample 4 vs. 5, 7 vs. 8; *P* > 0.05, sample 3 vs. 5, 6 vs. 8, Figures 6C,D).

These results suggested that when PHTPP suppresses the activity of ERβ in host cells, estrogen lacks its binding receptors in host cells infected with *N. meningitidis* isolates, and host cell inflammatory factors are upregulated. This suggested that estrogen plays a protective role in host cells infected with *N. meningitidis* by binding to ERβ.

Previous experimental results revealed that estrogen has a significant protective effect on *N. meningitidis* infection, and this effect is achieved by binding ERβ and downregulating the p38-MAPK signal pathway. Therefore, we aimed to further study whether the binding of estrogen and ERβ is involved in the p38-MAPK signal pathway. The results showed that when comparing the estrogen-*N. meningitidis* co-stimulation group and the PHTPP-estrogen-*N. meningitidis* co-stimulation group, the phosphorylation of p38 was significantly activated in the latter group (*P < 0.05, ^**^P < 0.01, ^***^P < 0.001, sample 4 vs. 5, 7 vs. 8, Figures 6E,F). Comparing the level of phosphorylated p38 in host cells between the *N. meningitidis* infection group and PHTPP-estrogen-*N. meningitidis* co-stimulation group, showed no significant difference in host cell level of phosphorylated p38 between the two groups (*P* > 0.05, sample 3 vs. 5, 6 vs. 8, Figures 6E,F).

These results suggested that when PHTPP suppresses the activity of ERβ, estrogen loses its binding receptors in *N. meningitidis*-infected host cells, loses its ability to inhibit the p38-MAPK pathway, and leads to the increased release of inflammatory factors in host cells infected by *N. meningitidis*.

## Discussion

*Neisseria meningitidis* is a bacteria that colonizes the mucosa of the human nasopharynx and could cause serious disease, such as septicemia and sepsis. The pathogenicity of *N. meningitidis* to host cells is closely related to the balance of the interaction between them, and the immune response of the body (Harrison et al., 2009; Zekas and Prossnitz, 2015; Bonazzi et al., 2018). As a steroid hormone, estrogen is involved in the immunomodulatory processes of many infectious diseases. It is not clear whether estrogen plays a role in *N. meningitidis* infection. In this study, we simulated the infection of *N. meningitidis* to HBMEC and MEC cell lines *in vitro*, and pretreated the cells with estrogen in advance. The results suggested that estrogen exerts a protective role on the cells during *N. meningitidis* infection. We found that estrogen could bind to estrogen receptor β (ERβ), regulate the p38-MAPK signaling pathway, and inhibits the release of inflammatory factors in host cells after *N. meningitidis* infection with TLR4 participating, thus reducing the occurrence and development of the inflammatory response of host cells after *N. meningitidis* infection.

Previous studies have shown that the severity and mortality of *N. meningitidis* disease are closely related to inflammatory cytokines produced by the host, such as IL-1, IL-6, IL-8, and TNF-α (Marriott et al., 2007; Raju et al., 2019). It has been confirmed that estrogen has different effects on different inflammatory responses (Holm et al., 2010; Farooq, 2015; Zeng et al., 2019). In the present study, we found that after *N. meningitidis* infected host cells, the release of inflammatory factors IL-6, IL-8, and TNF-α increased. Estrogen pretreatment could significantly reduce the release of these inflammatory factors caused by *N. meningitidis* infection, thus reducing the inflammatory response of the host cells. We inferred that estrogen plays a protective role in host cells in the process of *N. meningitidis* infection.

When we studied the decreased release of inflammatory factors from host cells after estrogen-mediated infection, we found that estrogen pretreatment inhibited the phosphorylation of MAPK downstream molecules induced by *N. meningitidis* infection and inhibited MAPK signaling pathway activation. As an important signaling pathway of innate immunity, the MAPK signaling pathway is very sensitive to the changes and metabolism of intracellular inflammatory factors. After activation, the MAPK signaling pathway plays an important role in the inflammatory response, cell proliferation, cell differentiation, apoptosis, cell invasion, and other reactions (Sprong et al., 2004; Holm et al., 2010; Farooq, 2015; Marshall et al., 2019). In *N. meningitidis*-infected cells, the MAPK signaling pathway was activated and the expression of IL-6, IL-8, and TNF-α increased. E2 inhibited the expression of inflammatory factors in host cells by inhibiting the activation of the MAPK signaling pathway, thereby reducing the inflammatory response of host cells and exerting a protective role on the host cells. Furthermore, we also found that E2 inhibits the MAPK signaling pathway by targeting p38.

The main biological response of p38-MAPK activation involves the production and activation of inflammatory mediators. Activated p38-MAPK positively regulates the expression of many inflammation-related genes, such as those encoding TNF-α, IL-1, IL-6, and IL-8. Previous studies have shown that SB203580, as a specific inhibitor of p38, could regulate and reduce the production of proinflammatory cytokines after inhibition of p38-MAPK (Frieling et al., 1997; Braun et al., 2002; Sprong et al., 2004; Li et al., 2017; Marshall et al., 2019). To verify the role of p38 in the E2 effect, we pretreated HBMECs and MECs infected with *N. meningitidis* with the p38 inhibitor SB203580. The results showed that inhibition of p38 activation by SB203580 could significantly reverse the downregulation of E2-mediated inflammatory factor release. When the p38-MAPK signaling pathway was blocked by SB203580, the protective effect of E2 on *N. meningitidis* infection disappeared. These results suggested that the protective effect of E2 on host cells depends on the p38-MAPK signaling pathway.

As the main virulence factor of *N. meningitidis*, LOS binds to a series of host transfer molecules and receptors of the innate immune system. LOS in the plasma and cerebrospinal fluid of patients with IMD is the main component of the inflammatory pathway activated by TLR4 (John et al., 2017). Our results showed that the protective role of E2 in *N. meningitidis* infection also depends on the participation of TLR4. When the specific shRNA for *TLR4* was used to inhibit the expression of TLR4, the downregulated inflammatory factors mediated by E2 showed a tendency of overexpression. These results suggested that the protective role of E2 in *N. meningitidis* infection is also targeted LOS-mediated TLR4 activation. These results improve our understanding of the molecular mechanism of the interaction between *N. meningitidi*s and host cells.

Estrogen acts by binding to its specific receptors α (ERα) and β (ERβ). We found that the level of ERβ in HBMECs and MECs was down-regulated after *N. meningitidis* infection, and the degree of down-regulation varied with the origin and serogroup of *N. meningitidis*. Invasive isolates and serogroup C invasive isolates were more likely to mediate the down-regulation of ERβ. The results are consistent with earlier finding that ERβ showed a down-regulation trend in infectious diseases; however, its expression was affected by pathogenic factors (different isolates) and can show a contradictory state in itself. The activation of the downstream MAPK signaling pathway by the binding of E2 and ERβ has been reported in many previous studies. In the study of *N. meningitidis* infection, we also found that E2 regulates the inflammatory response of host cells after infection through the ERβ-p38 MAPK-dependent signaling pathway. However, the deeply molecular mechanism of *N. meningitidis* down-regulating the expression of ERβ still needs further investigate.

In summary, we found that the host cell ERβ is downregulated to different extents by different isolates after *N. meningitidis* infection. Estrogen can inhibit the release of inflammatory factors induced by *N. meningitidis* infection and has a protective effect on host cells, which is achieved by regulating the ERβ-p38-MAPK signal pathway axis with TLR4 participating. These results reveal another possible explanation of the pathogenicity of *N. meningitidis* and provides a new direction to explore novel clinical treatment strategies for critically ill patients after *N. meningitidis* infection.

## Data Availability Statement

The original contributions presented in the study are included in the article/Supplementary Material, further inquiries can be directed to the corresponding author.

## Author Contributions

PG and JX conducted the study and wrote and revised the manuscript. HL conceived the idea. LX, WG, and ZC carried out the bacterial culture experiments. YG and MZ analyzed the data. GY helped revise the manuscript. ZS supervised the study.

## Conflict of Interest

The authors declare that the research was conducted in the absence of any commercial or financial relationships that could be construed as a potential conflict of interest.

## Publisher’s Note

All claims expressed in this article are solely those of the authors and do not necessarily represent those of their affiliated organizations, or those of the publisher, the editors and the reviewers. Any product that may be evaluated in this article, or claim that may be made by its manufacturer, is not guaranteed or endorsed by the publisher.
