# Supplementary material for: Estrogen Suppresses Cytokines Release in cc4821 Neisseria meningitidis Infection via TLR4 and ERβ-p38-MAPK Pathway
Source: Front Microbiol. 2022 Mar 29;13:834091. doi: 10.3389/fmicb.2022.834091 (PMC9002303; doi:10.3389/fmicb.2022.834091)
Supplement: Supplementary file 1 [file Data_Sheet_1.PDF]

Table 1 *N. meningitidis* isolates used in this study

Isolates ID	PubMLST ID	ST	cc	Serogroup	Source
320503	57870	4821	4821	C	Invasive isolates
340542	12672	4821	4821	C	Invasive isolates
100603	57853	4821	4821	C	Invasive isolates
341215	57852	4821	4821	B	Invasive isolates
321114	57867	3200	4821	B	Invasive isolates
370601	57868	3200	4821	C	Invasive isolates
431210	92589	4821	4821	B	Invasive isolates
330505	57871	4896	4821	C	Invasive isolates
340552	57856	4897	4821	B	Carried isolates
100572	57858	5610	4821	C	Carried isolates
100514	57860	4832	4821	C	Carried isolates
130803	57855	6928	4821	C	Carried isolates
421102	92487	3200	4821	B	Carried isolates
100503	NA[#]	4894	4821	C	Carried isolates
420703	92486	12311	4821	B	Carried isolates

320501	57869	4820	4821	C	Invasive isolates
420718	57862	11920	4821	C	Invasive isolates
440902	57852	4821	4821	B	Invasive isolates
440529	NA	7	5	A	Invasive isolates
130508	NA	7	5	A	Invasive isolates
310501	NA	7	5	A	Invasive isolates
510612	30466	7	5	A	Invasive isolates
100806	NA	2859	5	A	Invasive isolates
651801	NA	7	5	A	Invasive isolates
150720	NA	2146	198	cnl	Carried isolates
130817	72842	2146	198	cnl	Carried isolates
340809	NA	2146	198	cnl	Invasive isolates
211002	NA	2146	198	cnl	Carried isolates
341403	92583	4821	4821	C	Invasive isolates
341215	NA	4821	4821	B	Invasive isolates
LNT3	NA	7	5	A	Invasive isolates

440530	NA	7	5	A	Invasive isolates
421401	92586	12316	4821	B	Invasive isolates
421007	57861	4821	4821	B	Carried isolates
321102	92582	4821	4821	C	Invasive isolates

No genome sequence has been submitted to pubMLST.

Figure 1A Original gels of ERβ and β-actin

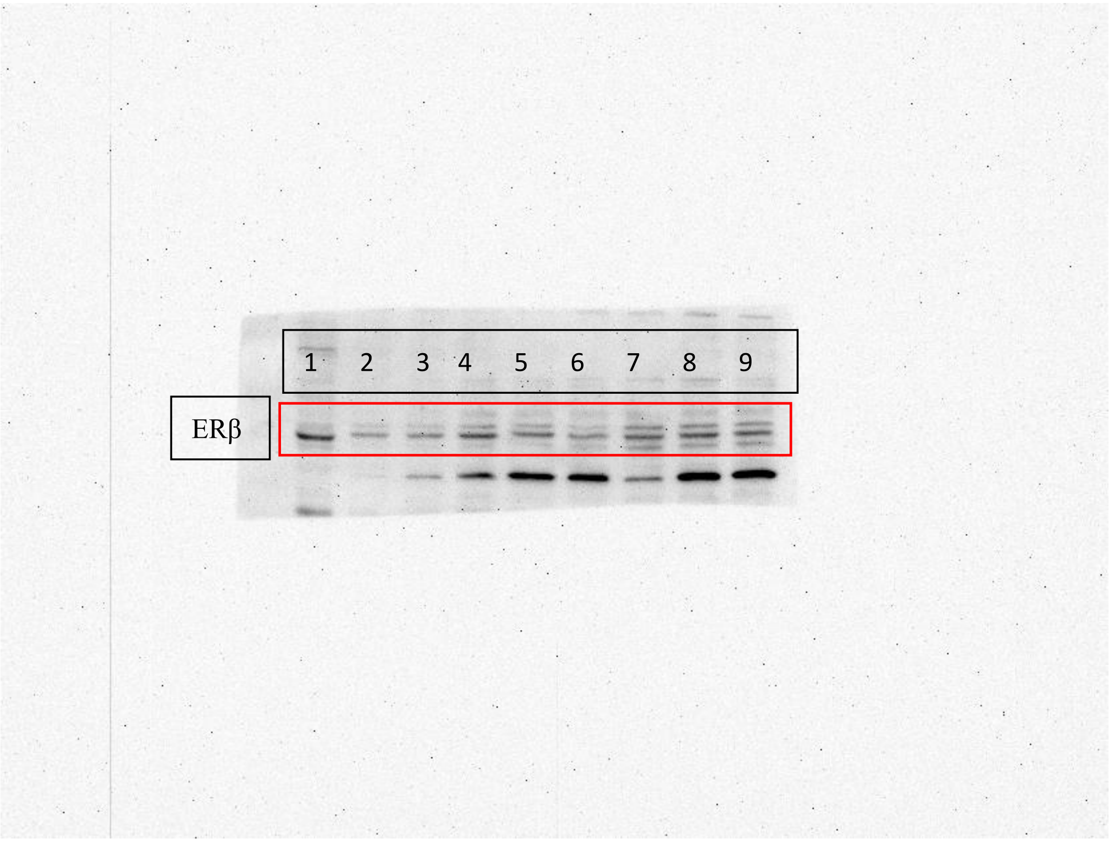

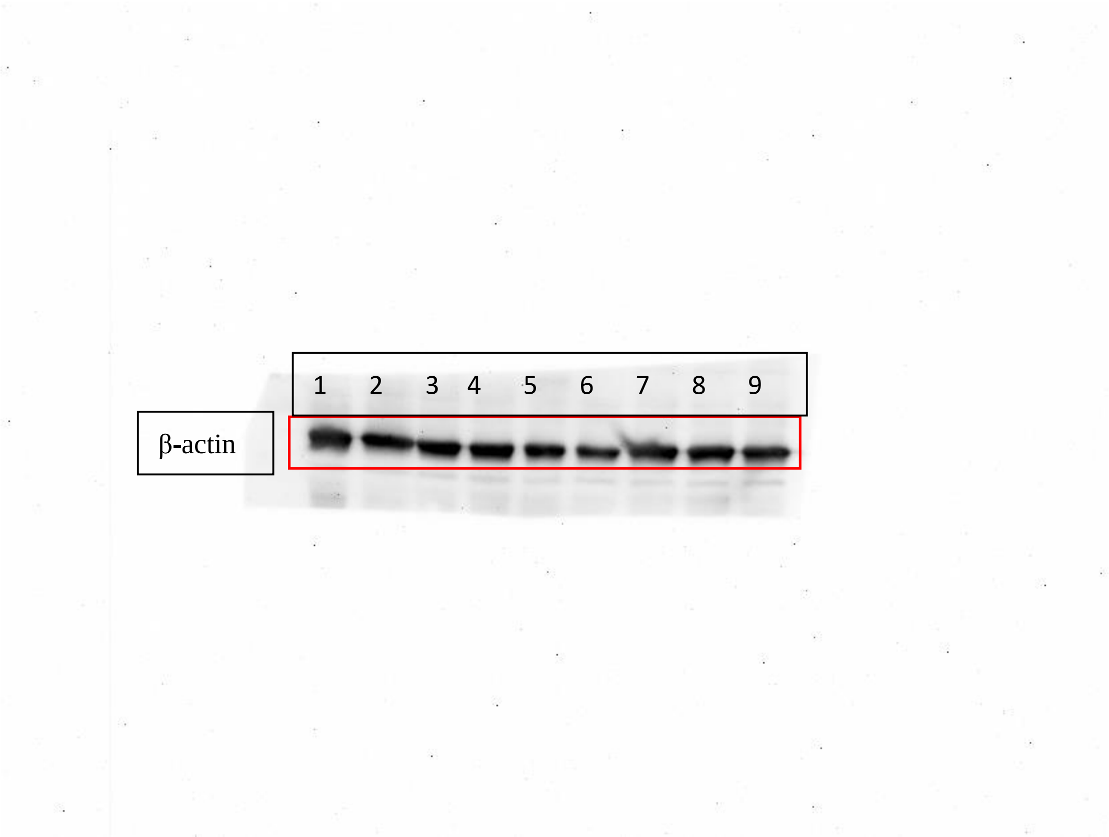

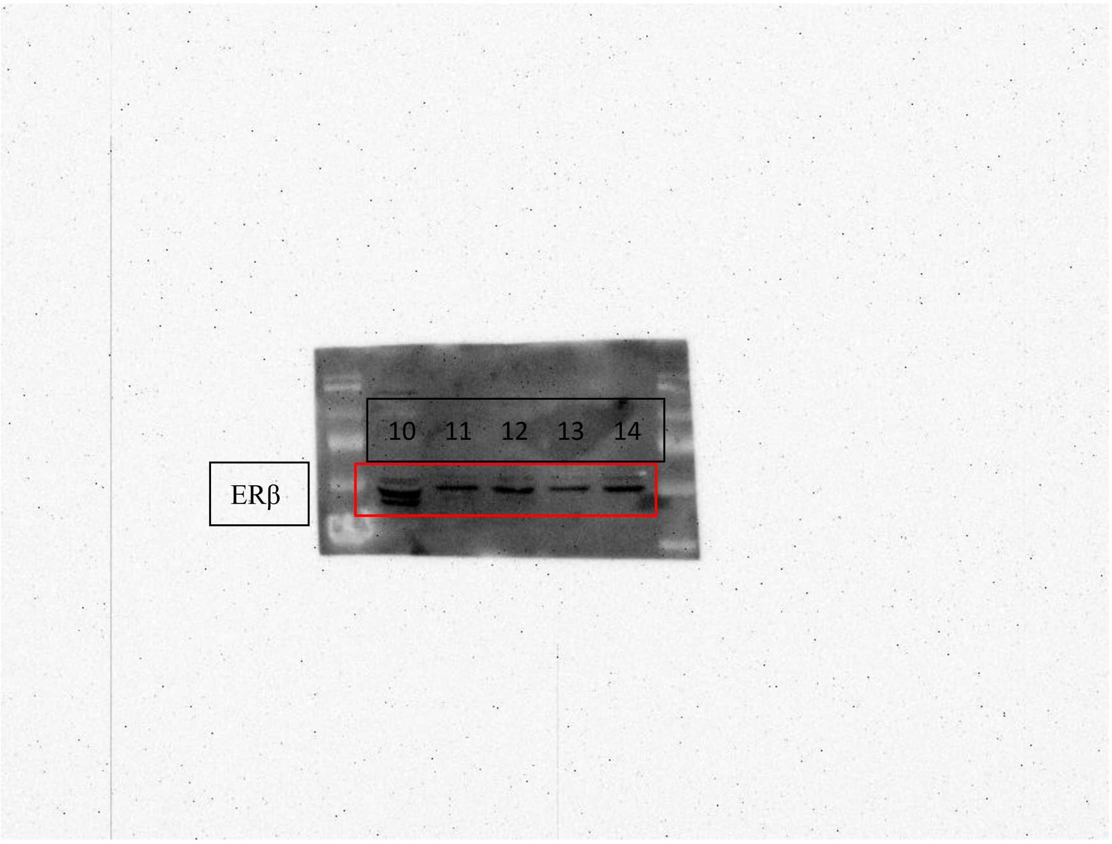
10 11 12 13 14
ERβ

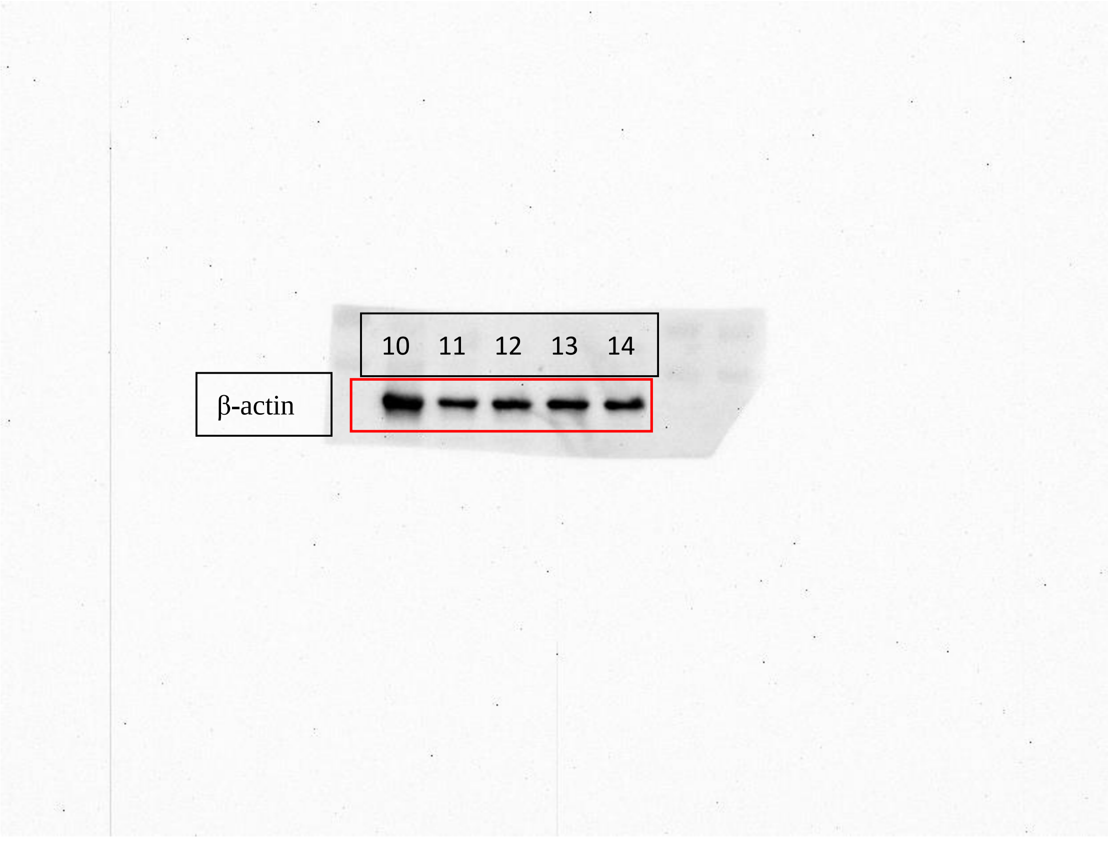
10 11 12 13 14
β-actin

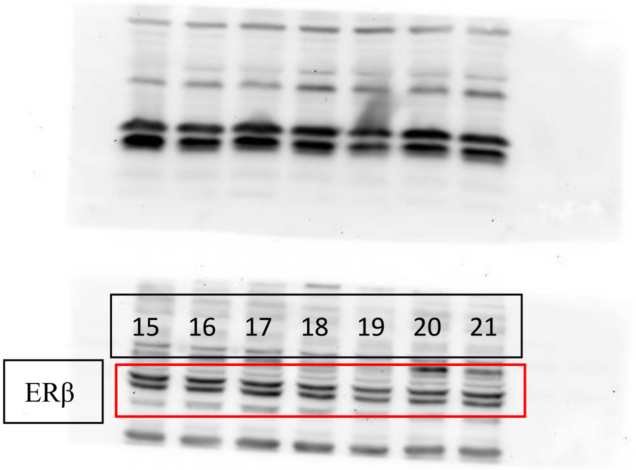
15 16 17 18 19 20 21
ERβ

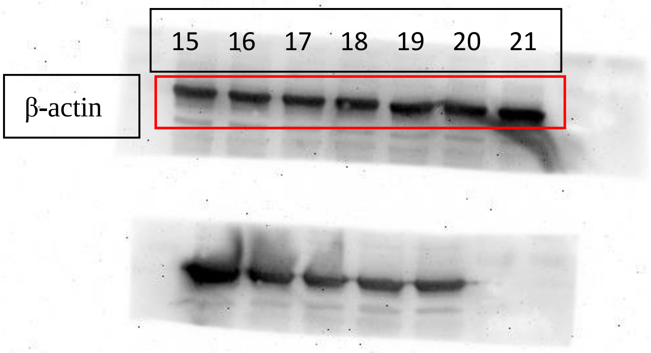
15 16 17 18 19 20 21
β-actin

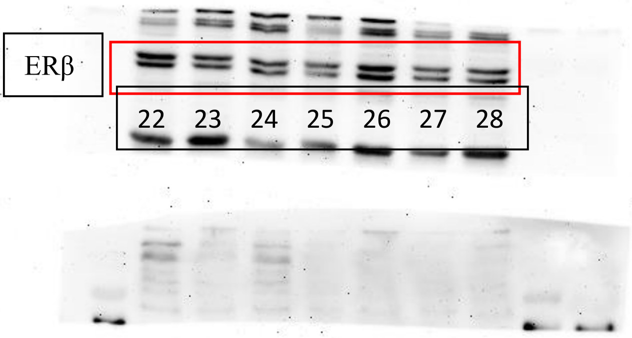
ERβ
22 23 24 25 26 27 28

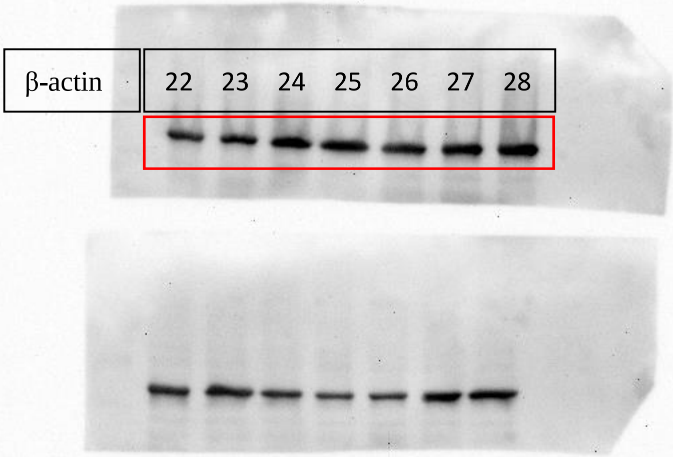
β-actin
22 23 24 25 26 27 28

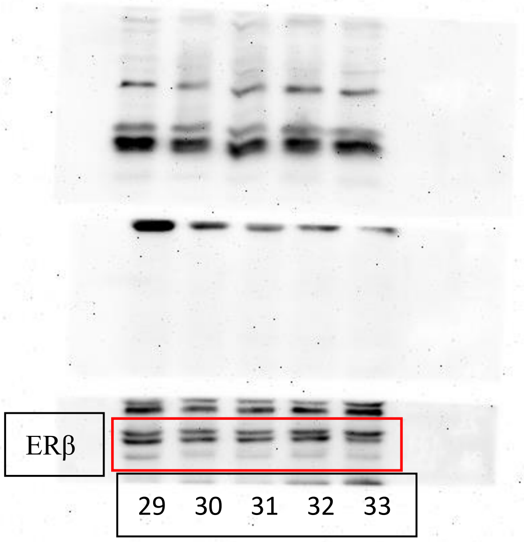
ERβ
29 30 31 32 33

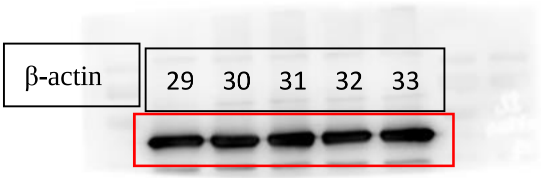
β-actin
29 30 31 32 33

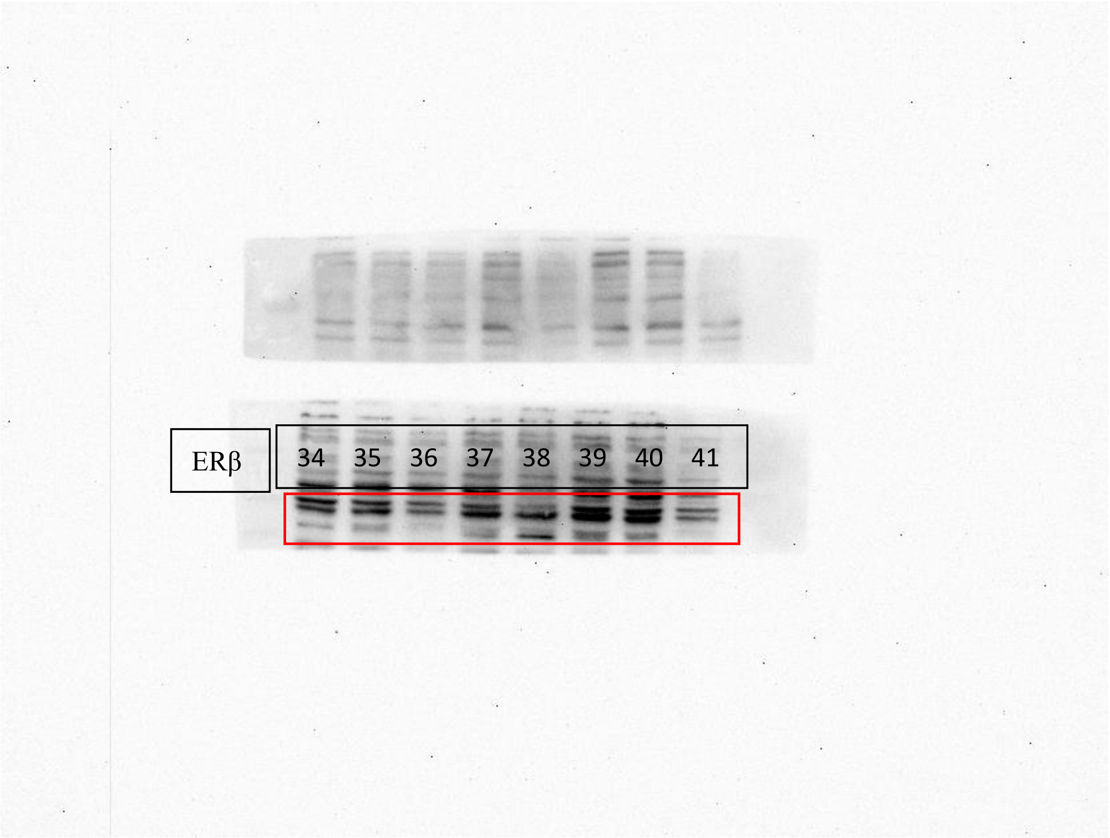
ERβ
34 35 36 37 38 39 40 41

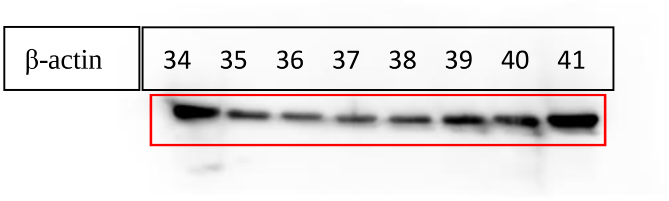
β-actin
34 35 36 37 38 39 40 41

Figure 1B Relative expression of ERβ in CTR group and *N. meningitidis* infection group ($\bar{x} \pm s$)

Experimental Group	CTR group	*N. meningitidis* infection group
ERβ expression/β-actin expression	1.00 ±0.08	0.62 ±0.28

Figure 1C Relative expression of ERβ in CTR group, carried isolates infection group and invasive isolates infection group ($\bar{x} \pm s$)

Experimental Group	CTR group	Carried isolates infection group	Invasive isolates infection group
ERβ expression/β-actin expression	1.00 ±0.08	0.88 ±0.16	0.52 ±0.24

Figure 1D Relative expression of ERβ in invasive isolates of different serogroup infection group ($\bar{x} \pm s$)

Experimental Group	MenA isolates infection group	MenB isolates infection group	MenC isolates infection group
ERβ expression/β-actin expression	0.58 ±0.15	0.47 ±0.15	0.36 ±0.11

Figure 2 A-B Inflammatory cytokines release in HBMEC and MEC cell lines

Cell lines	Experimental group	IL-6 (pg/mL)	IL-8 (pg/mL)	TNF-α (pg/mL)
HBMEC	CTR	6.96±0.50	4.92±0.77	1.79±0.08
	E2	6.37±0.21	5.08±0.19	1.74±0.04
	341215	369.64±16.48	563.50±56.44	51.12±6.92
	E2+341215	161.68±16.92	156.02±7.53	14.80±3.63
	340542	394.87±29.69	518.55±17.07	45.91±0.38
	E2+340542	118.79±19.17	147.77±19.27	13.49±1.97
	130803	133.86±5.36	293.96±24.51	27.39±3.25
	E2+130803	39.68±0.81	28.54±6.11	9.25±0.54
	360624	129.92±3.10	240.19±12.87	27.01±1.20
	E2+360624	23.60±1.62	25.15±2.67	5.63±0.92
MEC	CTR	6.59±0.29	4.44±0.28	2.03±0.17
	E2	6.03±0.21	4.14±0.28	2.02±0.35
	341215	433.11±21.07	540.78±40.70	56.47±4.36
	E2+341215	91.05±2.78	135.47±3.15	16.51±2.13
	340542	434.89±29.86	554.79±3.10	60.55±5.50
	E2+340542	89.27±5.42	141.12±5.51	13.74±1.03
	130803	197.37±24.26	193.13±12.83	36.20±3.56
	E2+130803	64.08±2.26	28.25±5.37	7.02±0.46
	360624	183.32±10.29	225.44±8.95	24.34±0.50
	E2+360624	44.38±0.90	39.58±1.38	5.99±0.34

Figure 2 C Original gel scanning of TNF-α and IL-6 in HBMEC cells

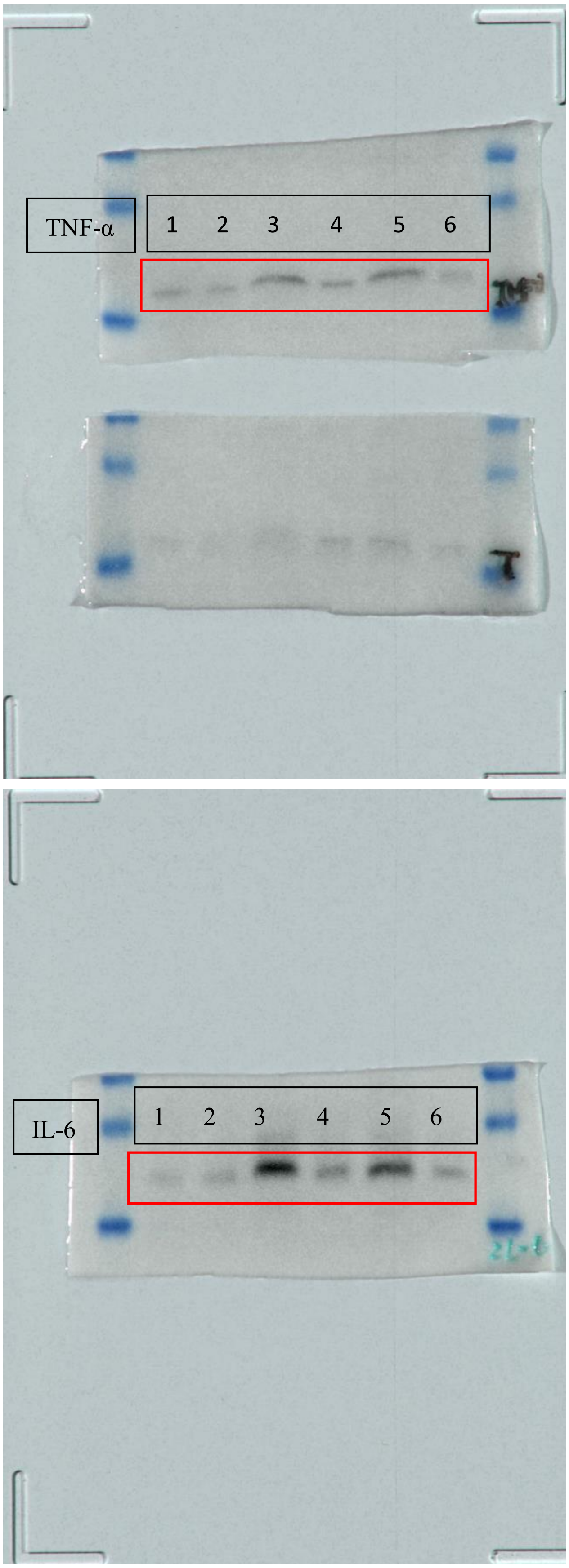

1. Control
2. E2
3. 341215
4. E2+341215
5. 340542
6. E2+340542

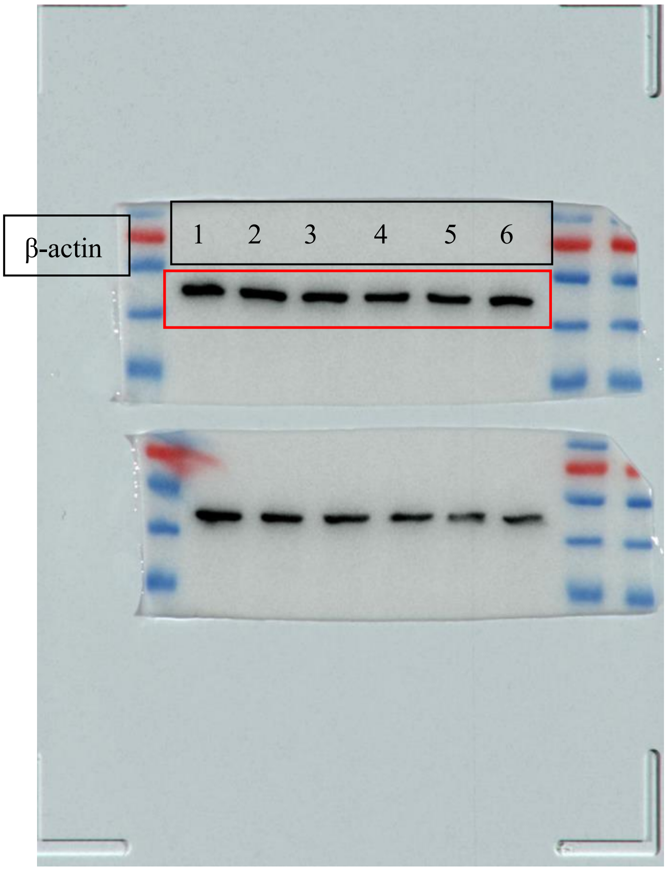

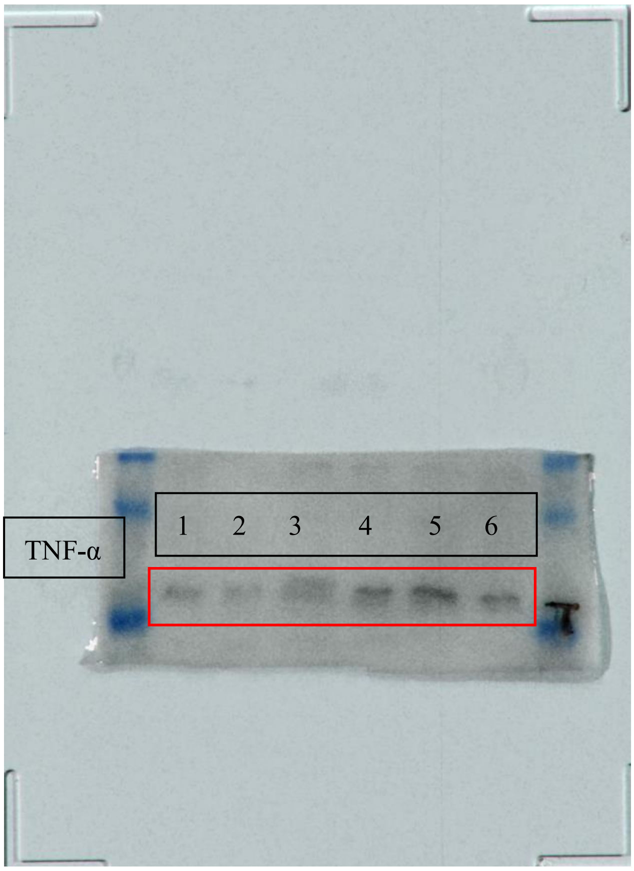

1. Control
2. E2
3. 130803
4. E2+130803
5. 360624
6. E2+360624

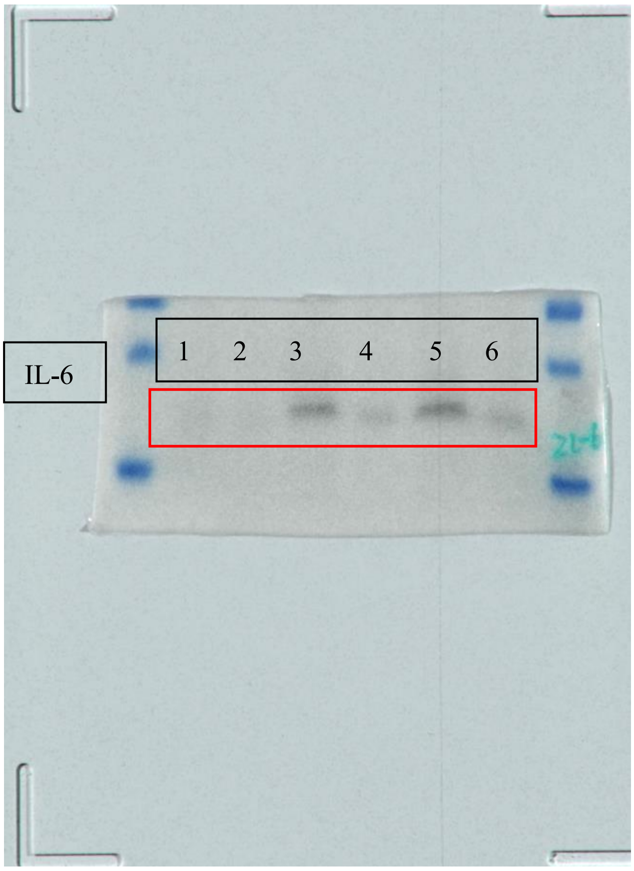
IL-6
1 2 3 4 5 6

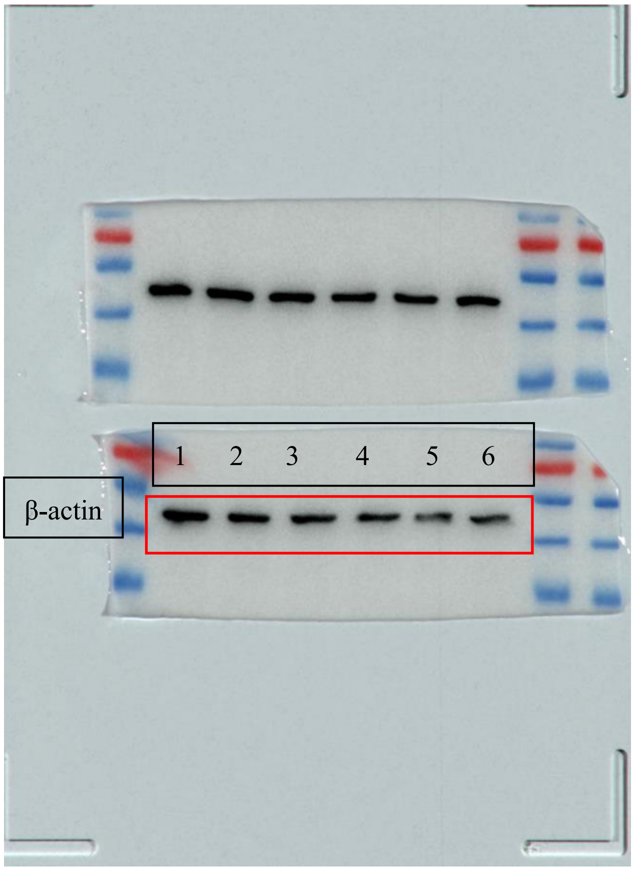
β-actin
1 2 3 4 5 6

Figure 2 D Original gel scanning of TNF-α and IL-6 in MEC cells

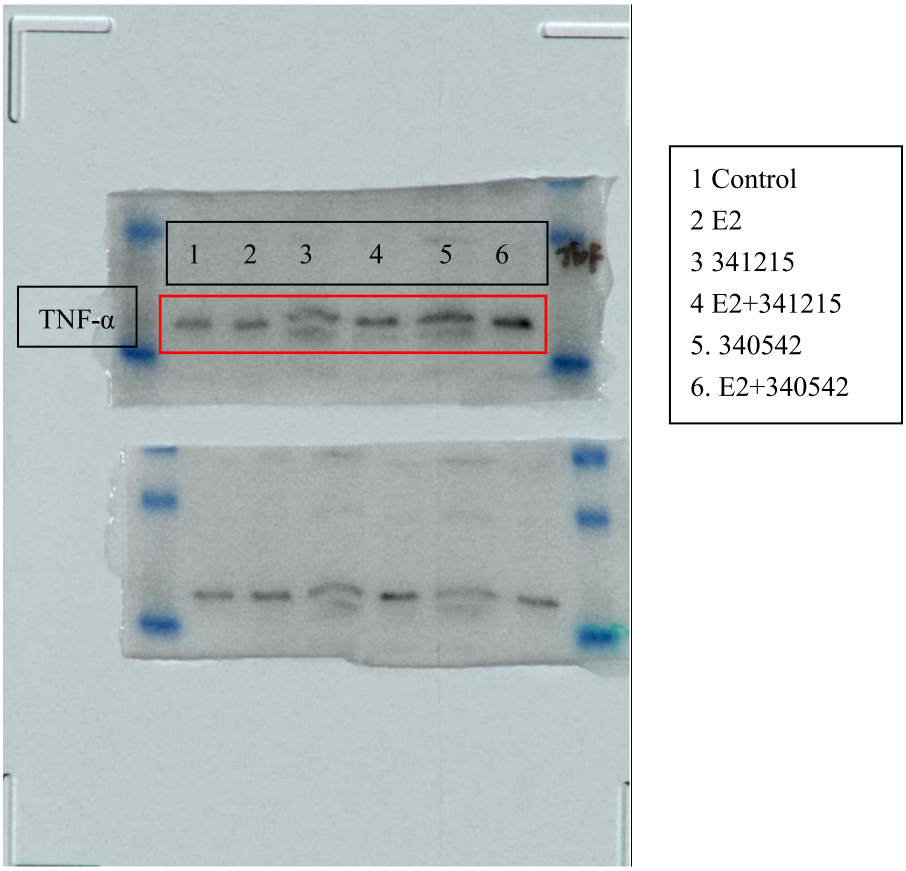

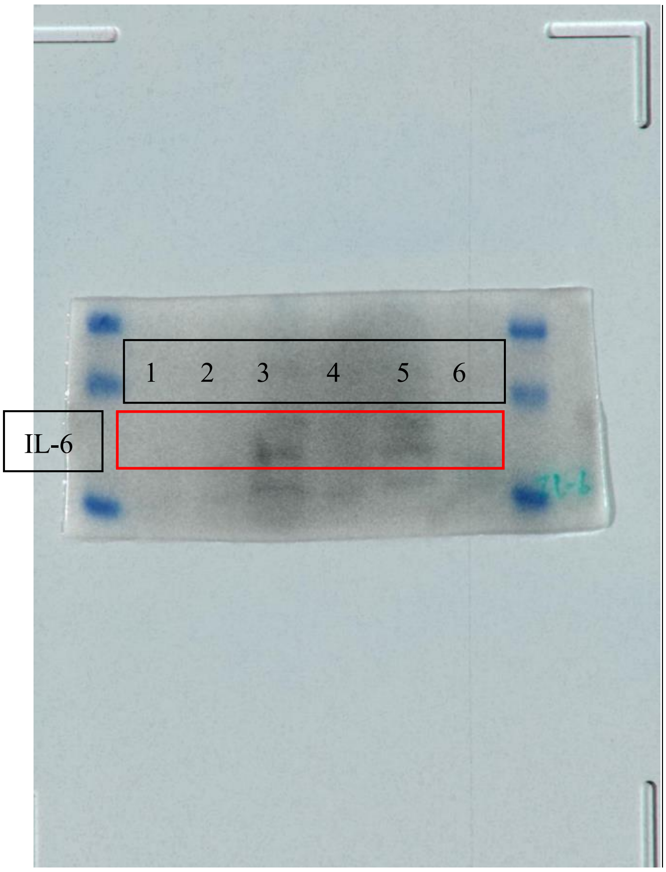

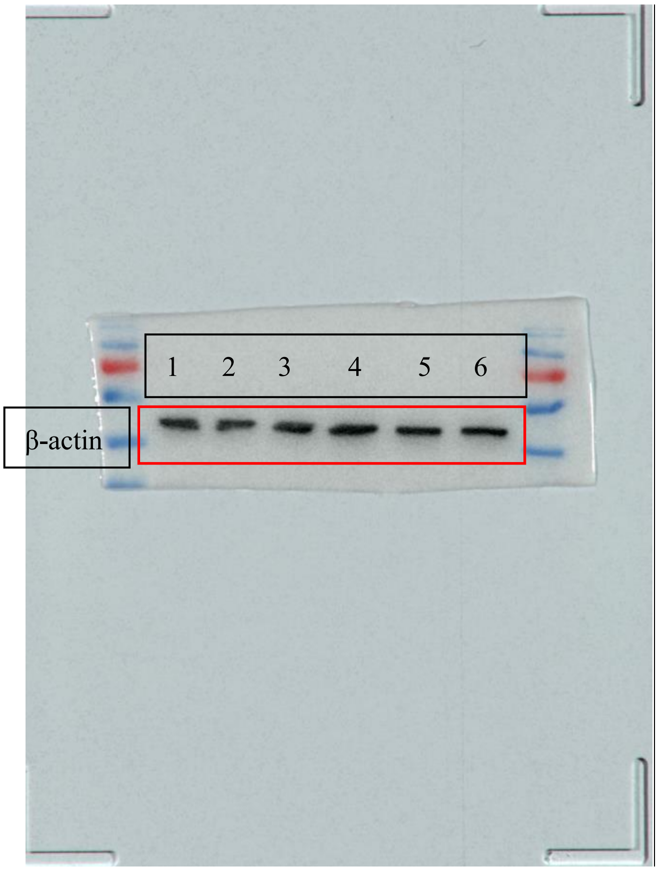

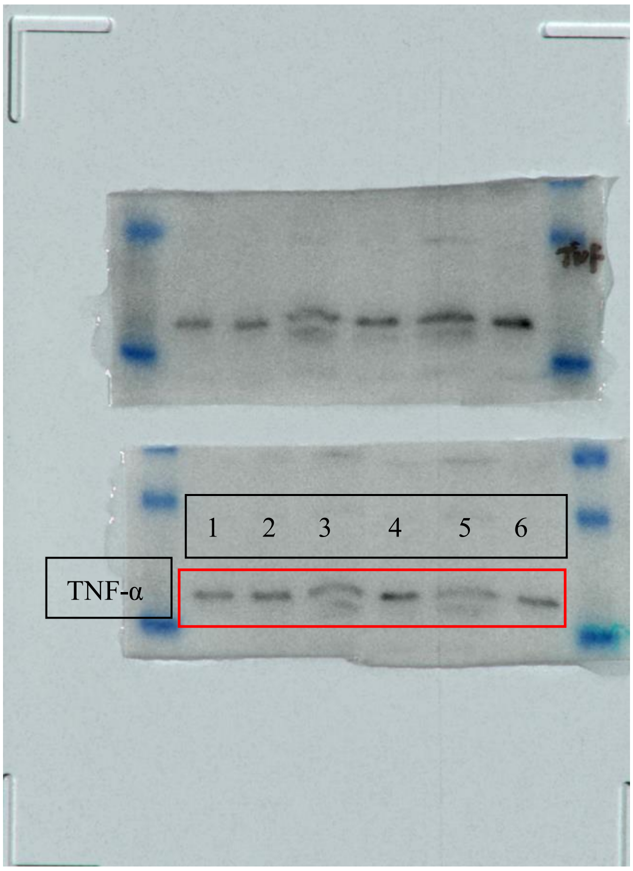

1. Control
2. E2
3. 130803
4. E2+130803
5. 360624
6. E2+360624

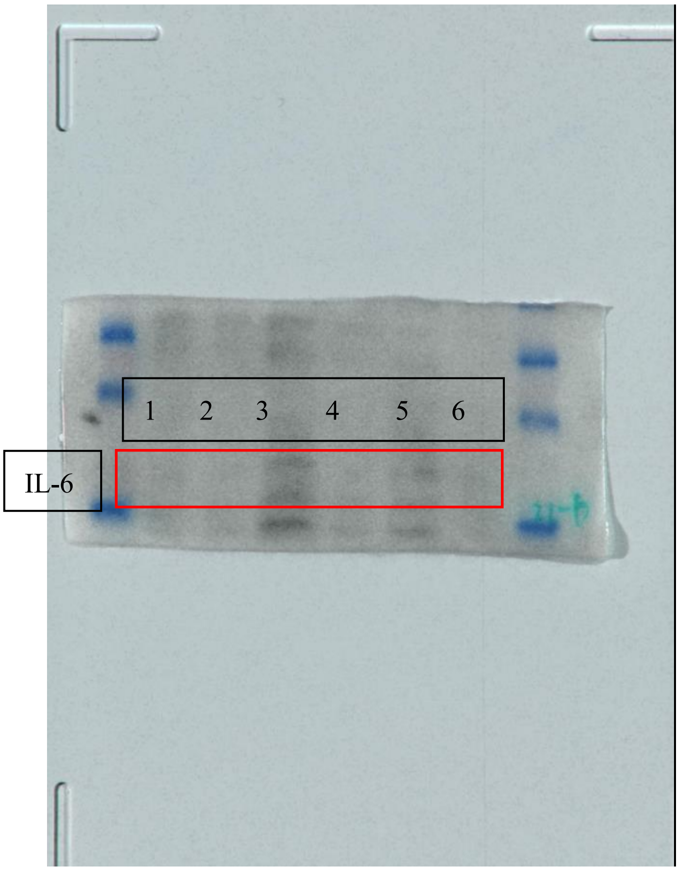

1 2 3 4 5 6
IL-6

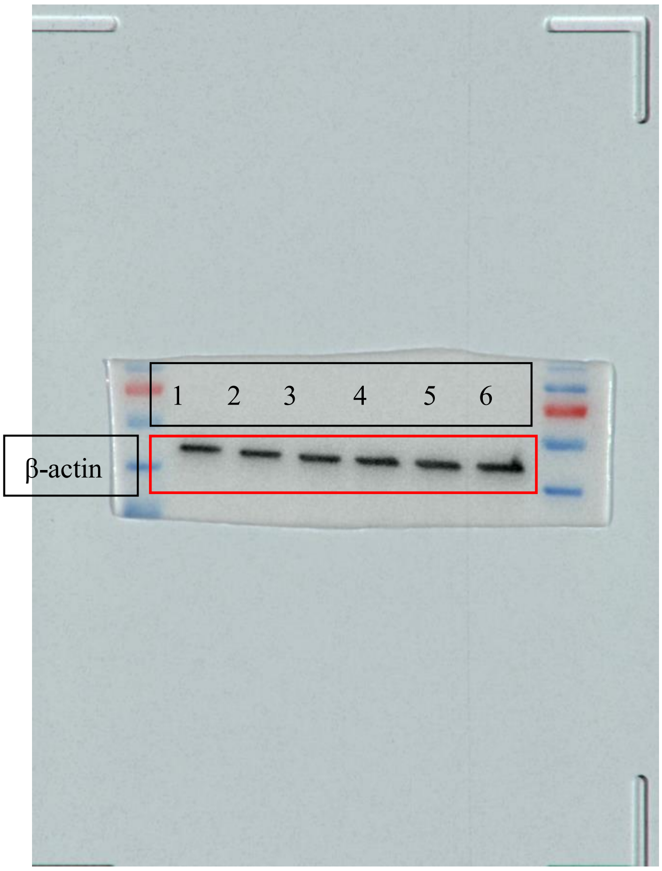

1 2 3 4 5 6
β-actin

Figure 2E-F Relative expression of TNF-α and IL-6 in HBMEC and MEC cell lines

Cell lines	Experimental group	TNF-α	IL-6
HBMEC	CTR	1.02±0.18	1.05±0.09
	E2	1.01±0.07	1.06±0.22
	341215	1.83±0.17	6.85±0.32
	E2+341215	1.07±0.10	1.36±0.19
	340542	2.07±0.05	4.43±0.48
	E2+340542	1.07±0.11	1.39±0.23
	CTR	1.01±0.02	1.03±0.06
	E2	0.92±0.14	0.93±0.07
	130803	1.58±0.04	2.85±0.14
	E2+130803	1.23±0.03	1.61±0.03
	360624	1.88±0.04	2.86±0.02
	E2+360624	1.06±0.04	1.42±0.13
MEC	CTR	1.00±0.10	1.01±0.08
	E2	1.01±0.06	1.01±0.13
	341215	1.53±0.09	1.60±0.08
	E2+341215	1.18±0.18	0.81±0.03
	340542	0.96±0.10	1.70±0.07
	E2+340542	1.75±0.05	0.85±0.02
	CTR	1.05±0.06	1.00±0.00
	E2	1.04±0.09	0.92±0.07
	130803	1.75±0.27	1.63±0.07
	E2+130803	1.09±0.14	0.89±0.01
	360624	0.85±0.01	1.35±0.15
	E2+360624	0.99±0.10	0.76±0.05

Figure 3A Original gel scanning

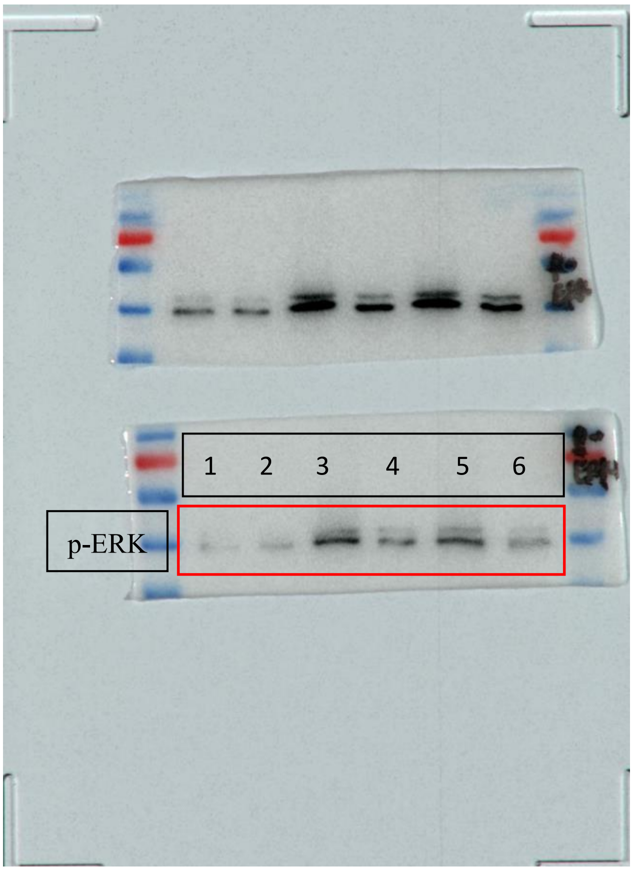

1. Control
2. E2
3. 341215
4. E2+341215
5. 340542
6. E2+340542

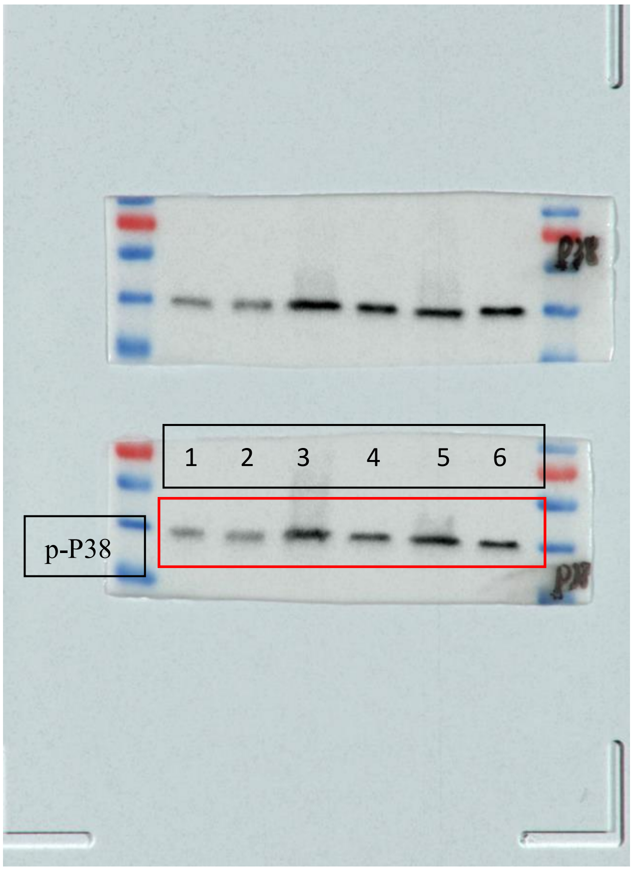

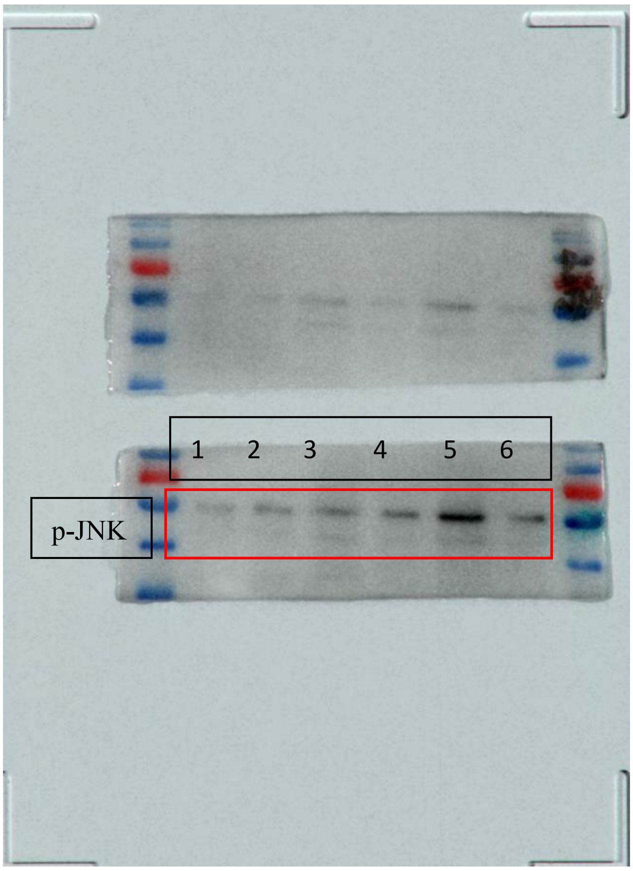
1 2 3 4 5 6
p-JNK

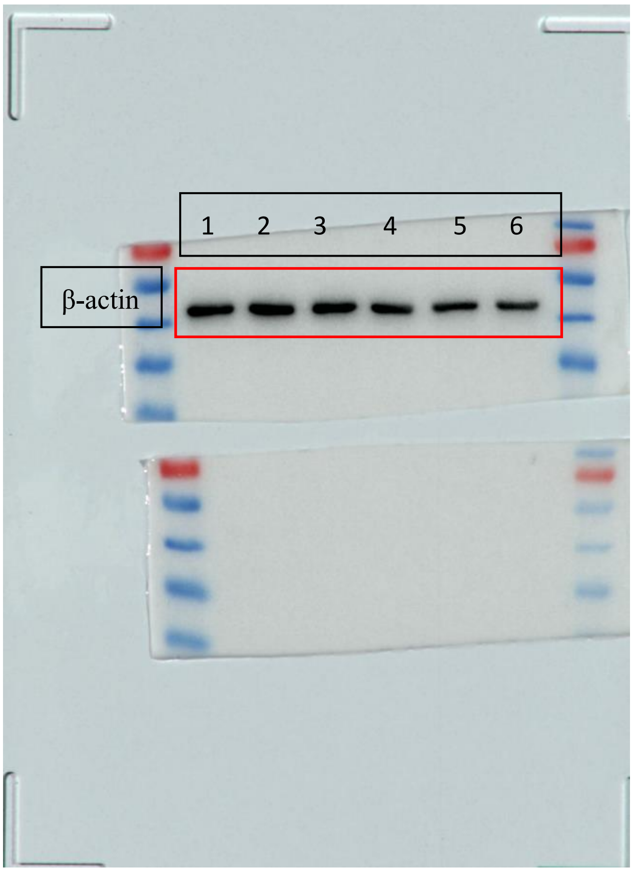
1 2 3 4 5 6
β-actin

Figure 3B Original gel scanning

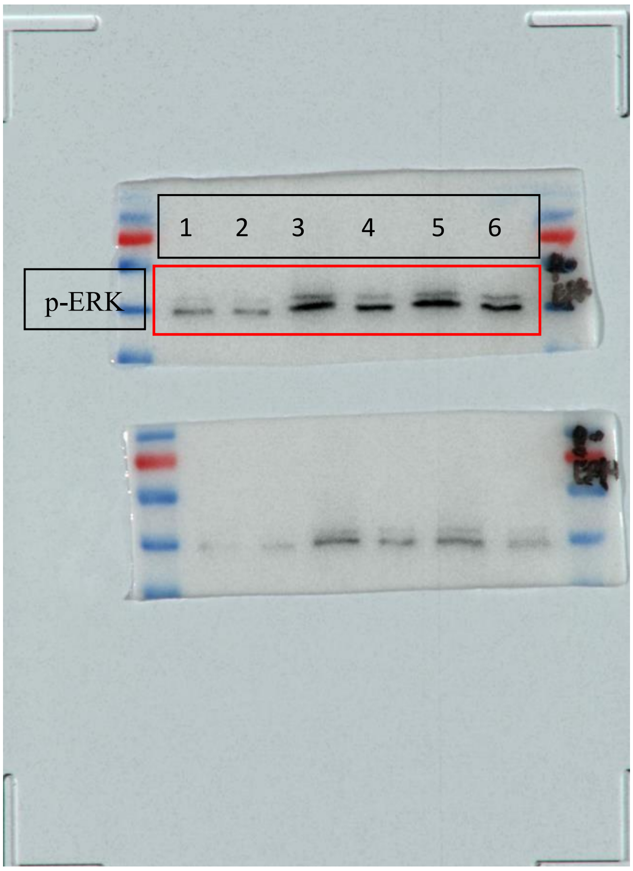

1. Control
2. E2
3. 130803
4. E2+130803
5. 360624
6. E2+360624

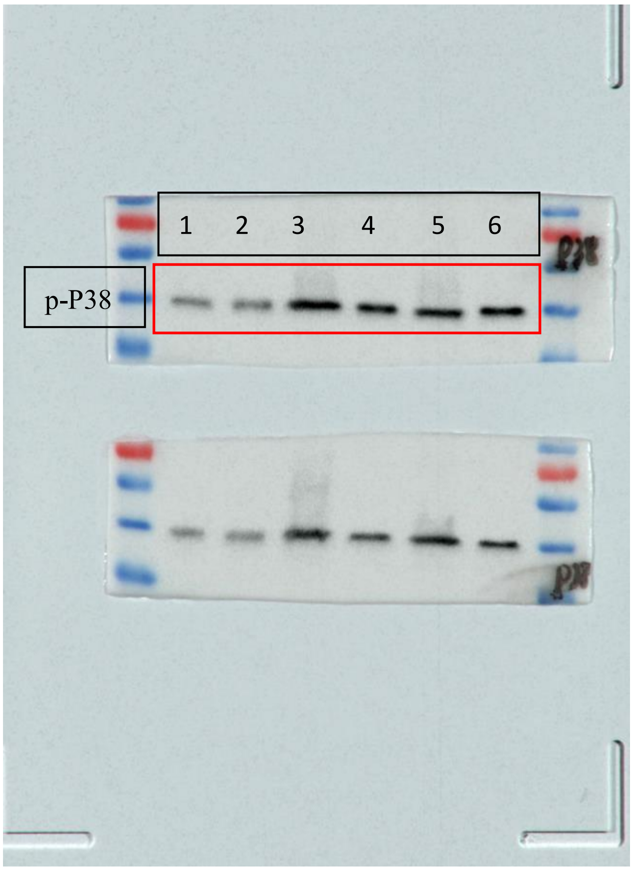

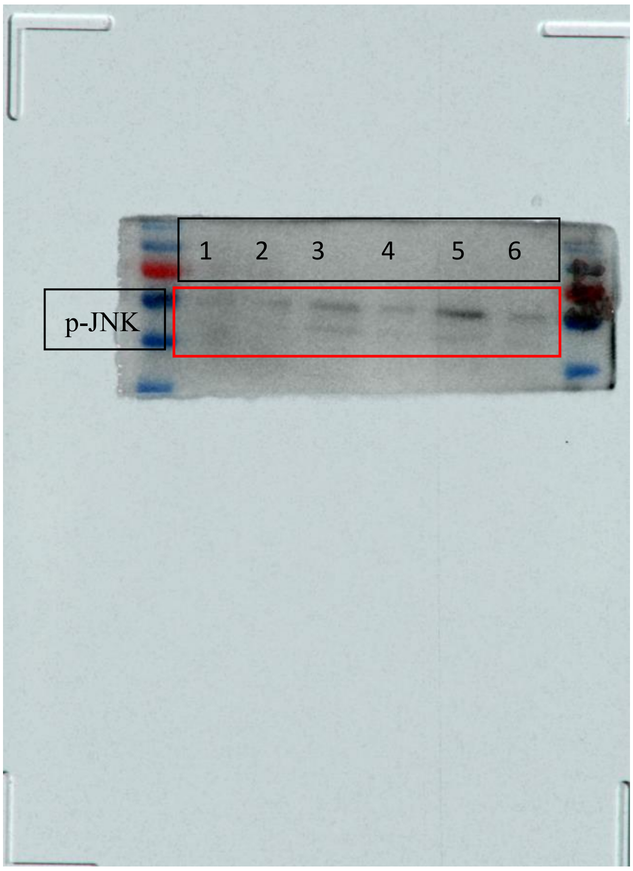
1 2 3 4 5 6
p-JNK

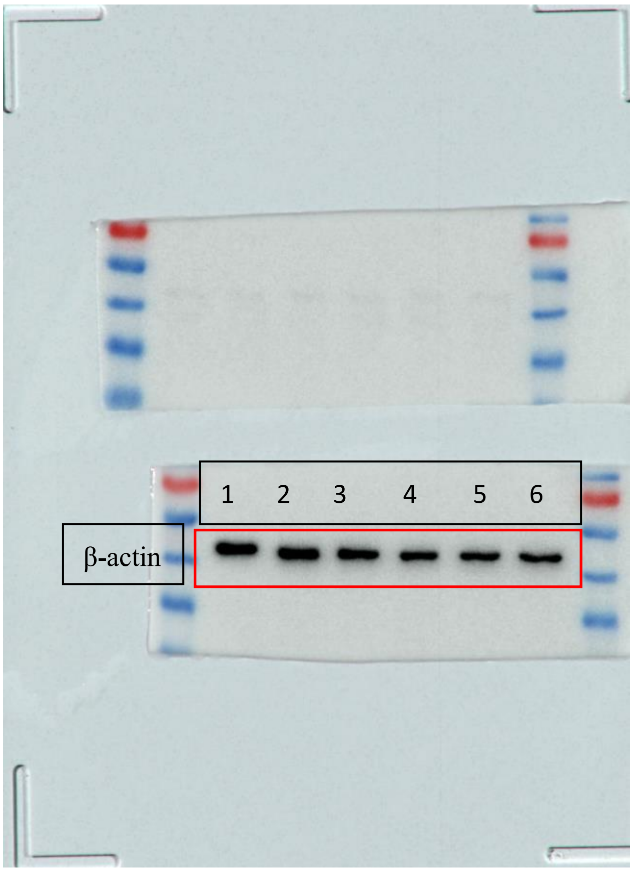
1 2 3 4 5 6
β-actin

Figure 3C-D Relative expression of p-ERK, p-JNK and p-P38 in HBMEC cells

Cell lines	Experimental group	p-ERK	p-JNK	p-P38
HBMEC	CTR	1.00±0.00	1.00±0.00	1.00±0.00
	E2	1.00±0.00	1.14±0.14	1.04±0.01
	341215	4.51±0.20	4.38±0.16	1.49±0.04
	E2+341215	1.23±0.12	2.52±0.05	1.00±0.00
	340542	3.67±0.15	3.75±0.10	5.55±0.02
	E2+340542	1.00±0.00	2.60±0.27	1.00±0.00
	CTR	1.07±0.09	1.01±0.02	1.01±0.01
	E2	0.17±0.16	1.00±0.01	1.01±0.02
	130803	5.91±0.13	5.55±0.06	1.60±0.06
	E2+130803	3.69±0.04	3.56±0.12	1.00±0.00
	360624	5.68±0.05	5.62±0.21	2.73±0.15
	E2+360624	3.06±0.35	4.60±0.14	1.01±0.02

Figure 3E Original gel scanning

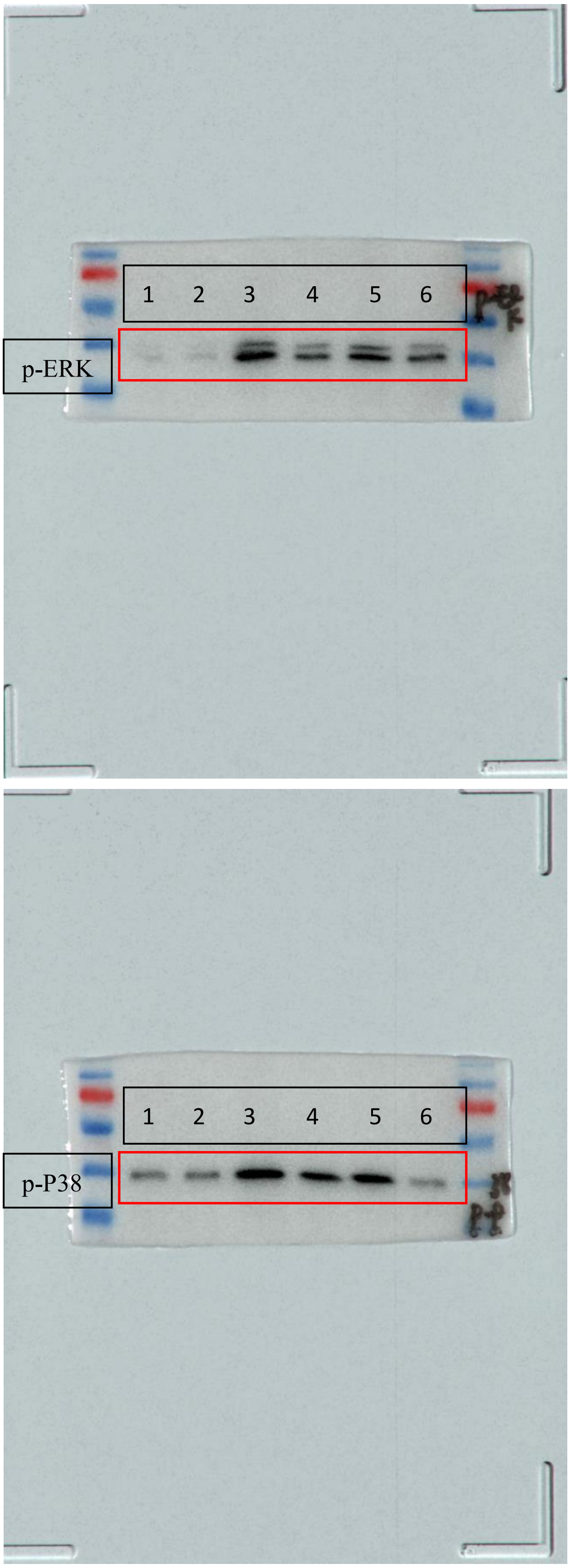

1. Control
2. E2
3. 341215
4. E2+341215
5. 340542
6. E2+340542

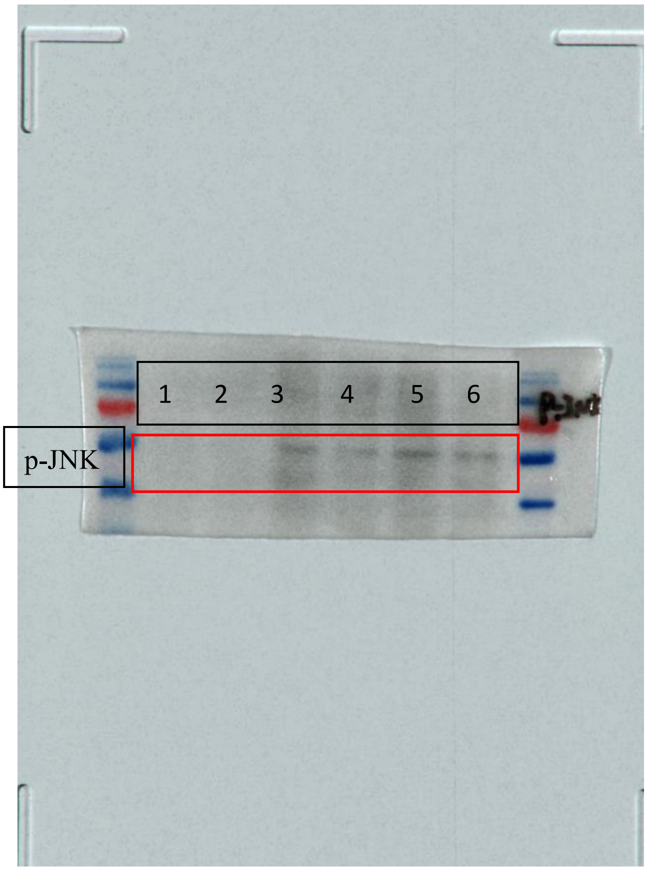
1 2 3 4 5 6
p-JNK

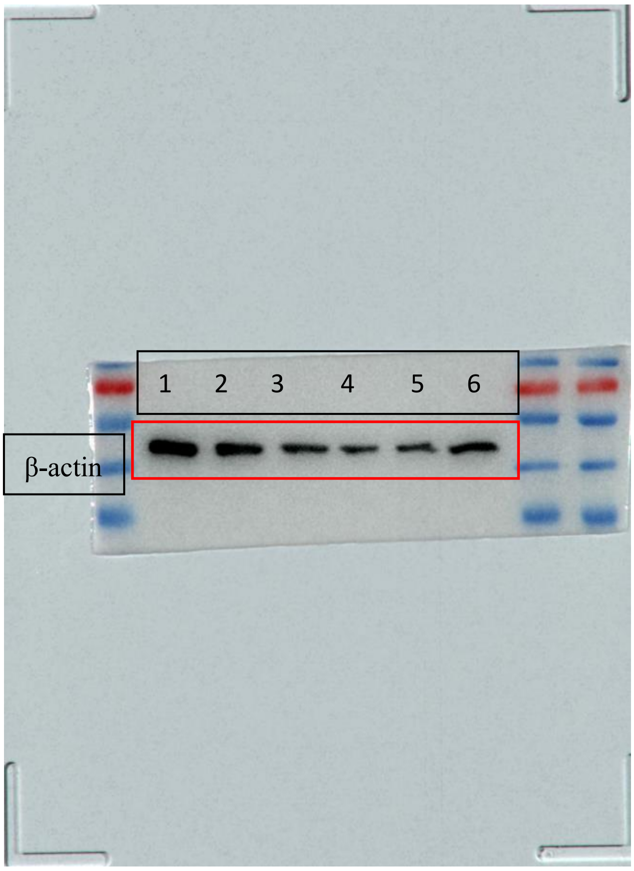
1 2 3 4 5 6
β-actin

Figure 3F Original gel scanning

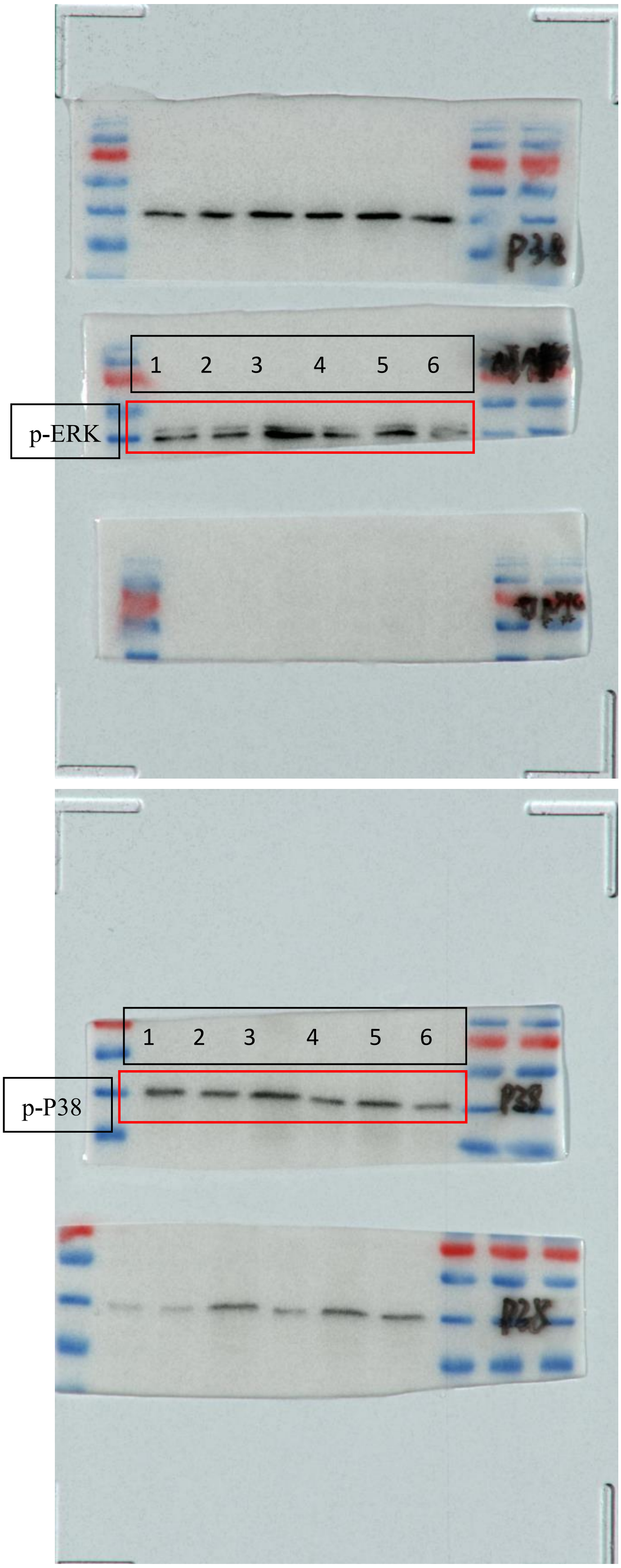

1. Control
2. E2
3. 130803
4. E2+130803
5. 360624
6. E2+360624

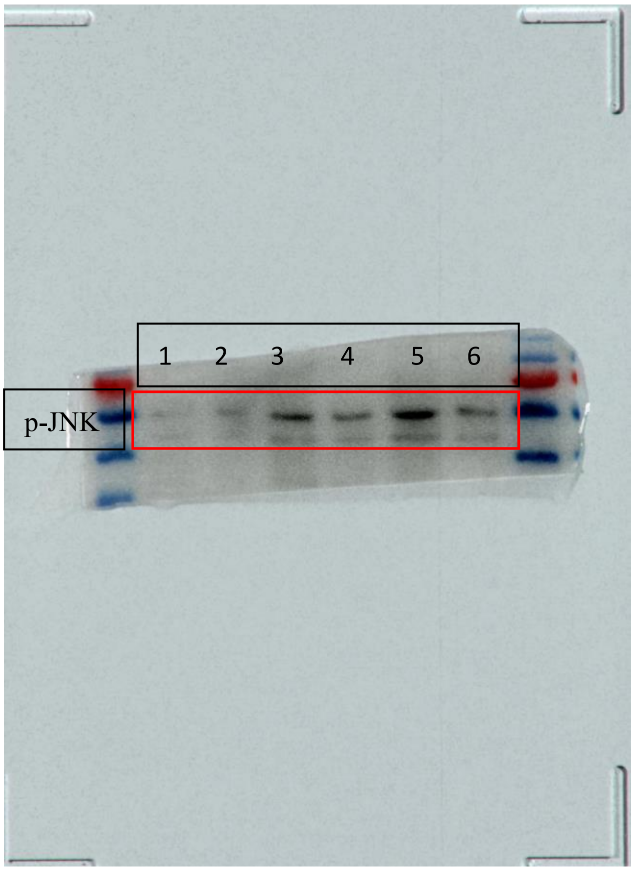
1 2 3 4 5 6
p-JNK

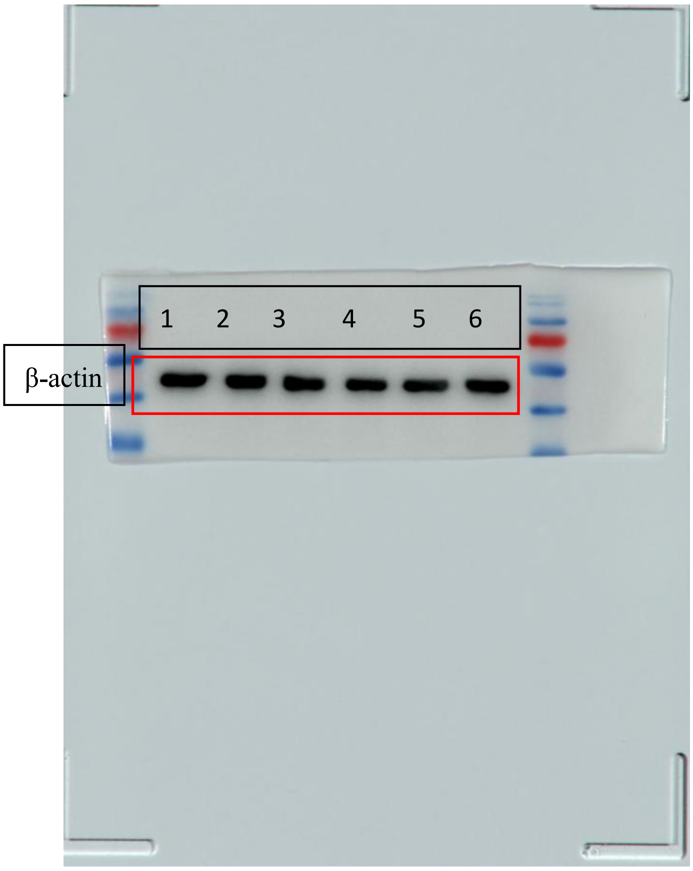
1 2 3 4 5 6
β-actin

Figure 3G-H Relative expression of p-ERK, p-JNK and p-P38 in MEC cells

Cell lines	Experimental group	p-ERK	p-JNK	p-P38
MEC	CTR	1.00±0.08	1.00±0.01	1.01±0.05
	E2	1.06±0.05	0.99±0.02	1.03±0.06
	341215	7.53±0.19	3.55±0.08	2.60±0.06
	E2+341215	3.38±0.12	1.84±0.04	1.51±0.05
	340542	6.59±0.2	3.09±0.05	2.64±0.01
	E2+340542	3.30±0.38	0.93±0.02	1.64±0.09
	CTR	0.99±0.01	1.00±0.01	1.00±0.00
	E2	0.96±0.01	0.98±0.02	1.03±0.02
	130803	2.66±0.12	2.74±0.01	3.49±0.04
	E2+130803	0.94±0.04	0.97±0.03	1.54±0.04
	360624	1.51±0.05	2.42±0.10	3.96±0.04
	E2+360624	0.70±0.08	0.88±0.08	1.66±0.08

Figure 4A-D Original gel scanning and relative expression data

A. Original gel scanning in HBMEC cells

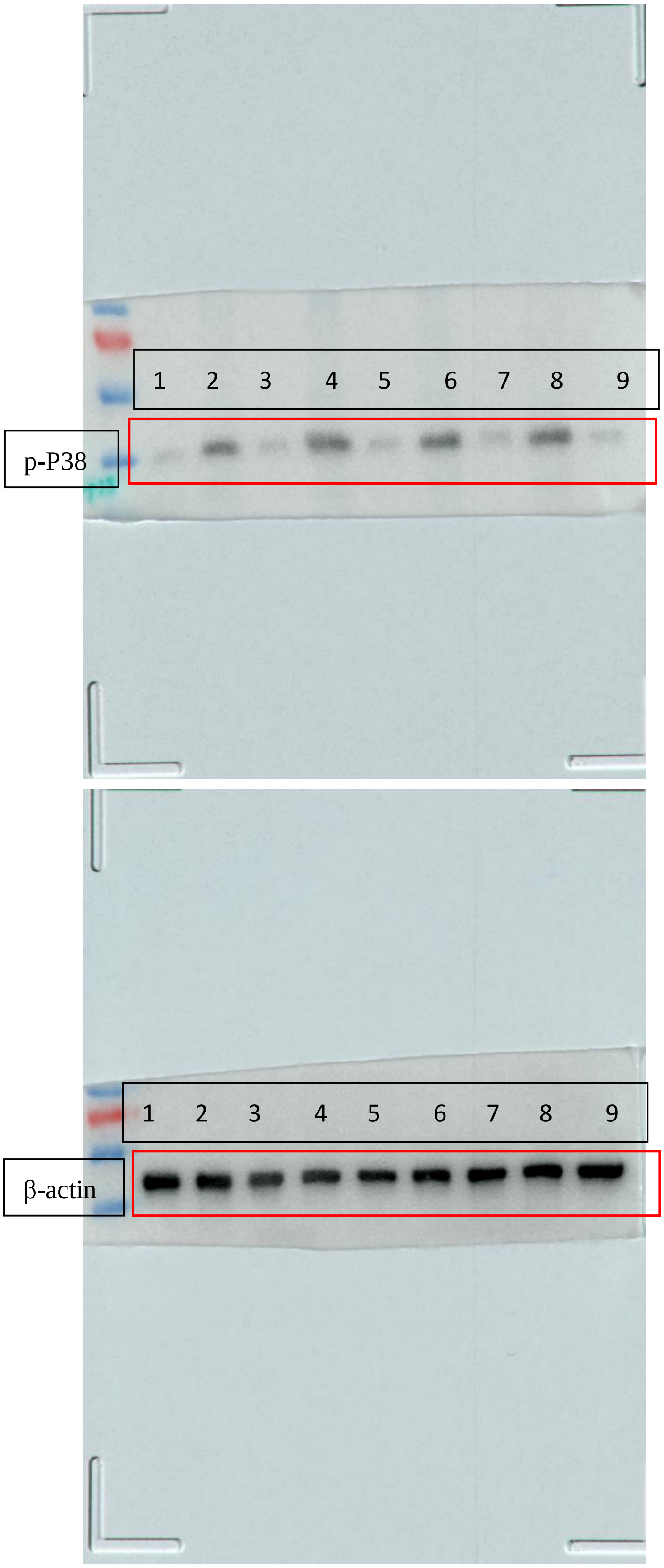

1. Control
2. 341215
3. 341215+SB203580
4. 340542
5. 340542+SB203580
6. 130803
7. 130803+SB203580
8. 360624
9. 360624+SB203580

C. Original gel scanning in MEC cells

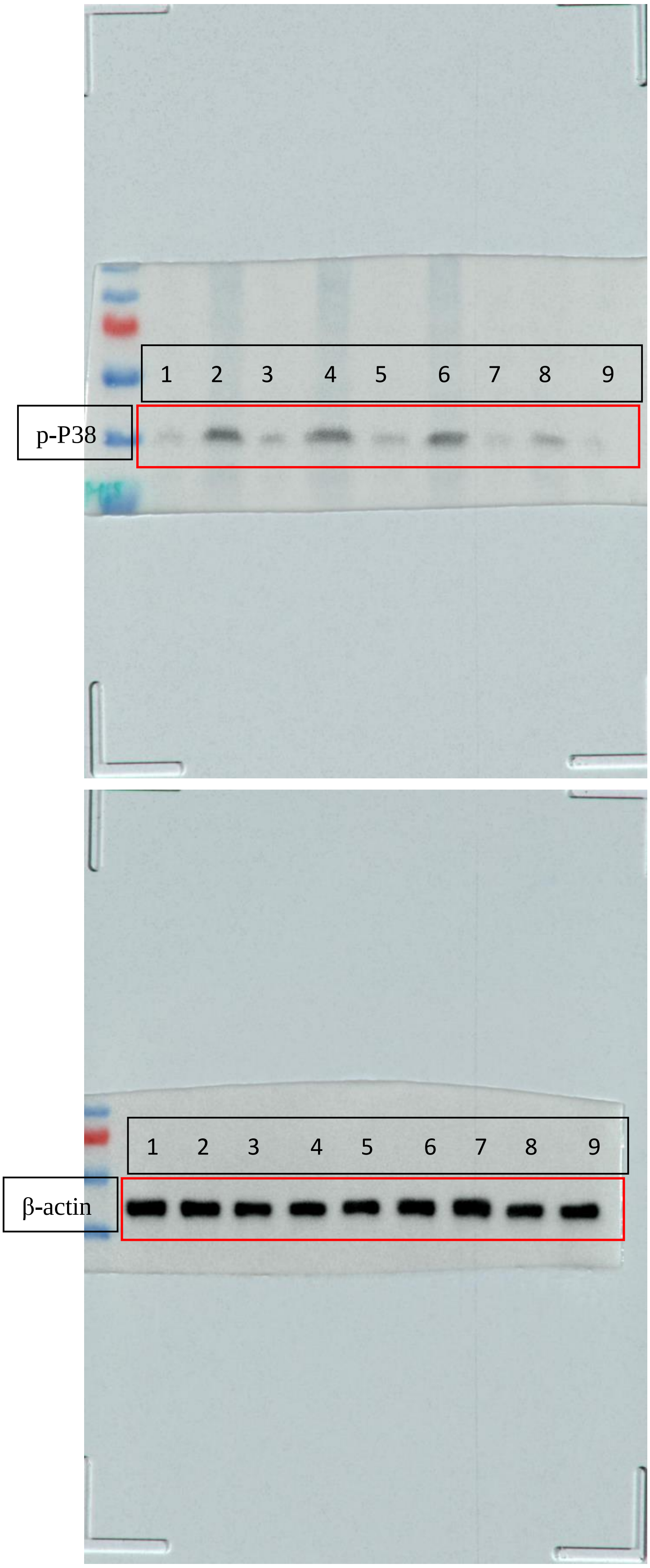

1. Control
2. 341215
3. 341215+SB203580
4. 340542
5. 340542+SB203580
6. 130803
7. 130803+SB203580
8. 360624
9. 360624+SB203580

B, D. Relative expression of p-P38

Cell lines	Experimental group	Relative expression
HBMEC	CTR	1.00±0.02
	341215	2.89±0.19
	341215 + SB203580	1.16±0.21
	340542	3.15±0.22
	340542 + SB203580	0.85±0.01
	130803	2.58±0.07
	130803 + SB203580	0.87±0.04
	360624	2.72±0.15
	360624 + SB203580	1.10±0.15
MEC	CTR	1.00±0.06
	341215	3.32±0.02
	341215 + SB203580	1.39±0.09
	340542	2.67±0.30
	340542 + SB203580	0.92±0.03
	130803	3.36±0.04
	130803 + SB203580	0.92±0.09
	360624	1.30±0.17
	360624 + SB203580	0.63±0.03

Figure 4E-F. Inflammatory cytokines release in HBMEC and MEC cell lines

Cell lines	Experimental groups	Inflammatory cytokines（pg/ml）		
		IL-6	IL-8	TNF-α
HBMEC	E2+341215+SB203580	355.46±15.09	581.58±41.91	52.13±7.67
HBMEC	E2+340542+SB203580	359.68±52.29	519.90±35.62	44.41±2.44
HBMEC	E2+130803+SB203580	127.21±23.62	277.00±19.97	26.23±3.00
HBMEC	E2+360624+SB203580	134.69±4.89	245.91±17.61	27.26±1.25
MEC	E2+341215+SB203580	438.08±17.35	538.12±29.17	55.84±2.72
MEC	E2+340542+SB203580	435.50±29.36	548.90±22.40	60.04±0.33
MEC	E2+130803+SB203580	204.76±32.68	195.11±17.13	38.17±3.44
MEC	E2+360624+SB203580	183.87±21.37	227.22±19.55	23.34±4.07

Figure 4G-I Original gel scanning and relative expression data

G. Original gel scanning in HBMEC cells

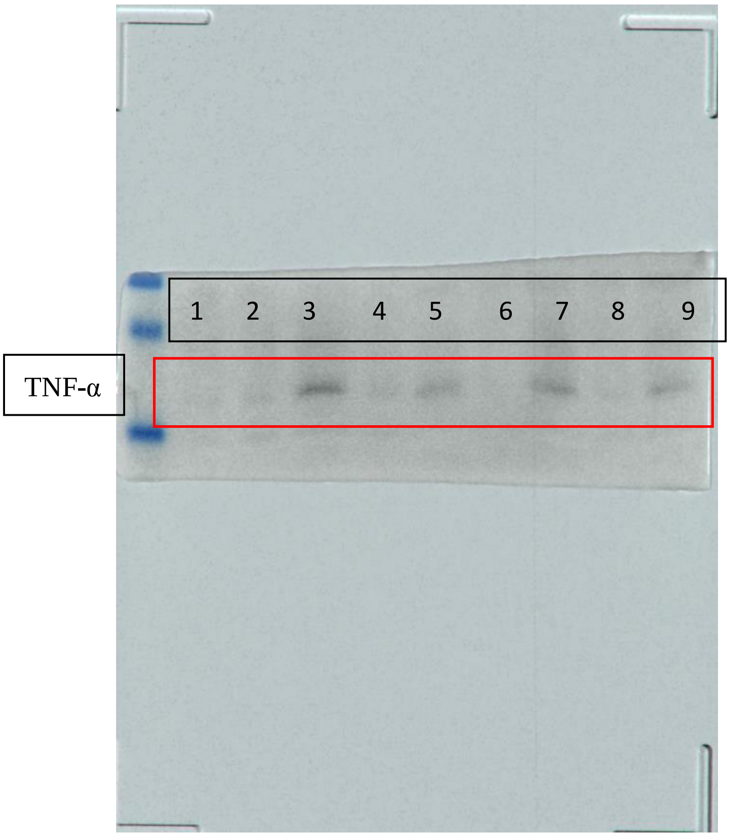

1. Control
2. E2+341215
3. E2+341215+SB203580
4. E2+340542
5. E2+340542+SB203580
6. E2+130803
7. E2+130803+SB203580
8. E2+360624
9. E2+360624+SB203580

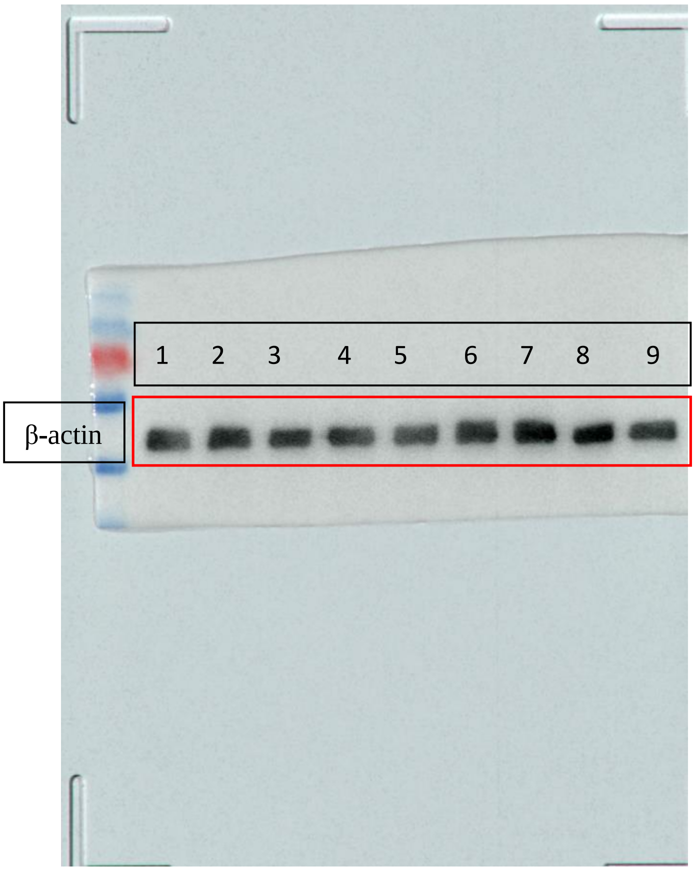

I. Original gel scanning in MEC cells

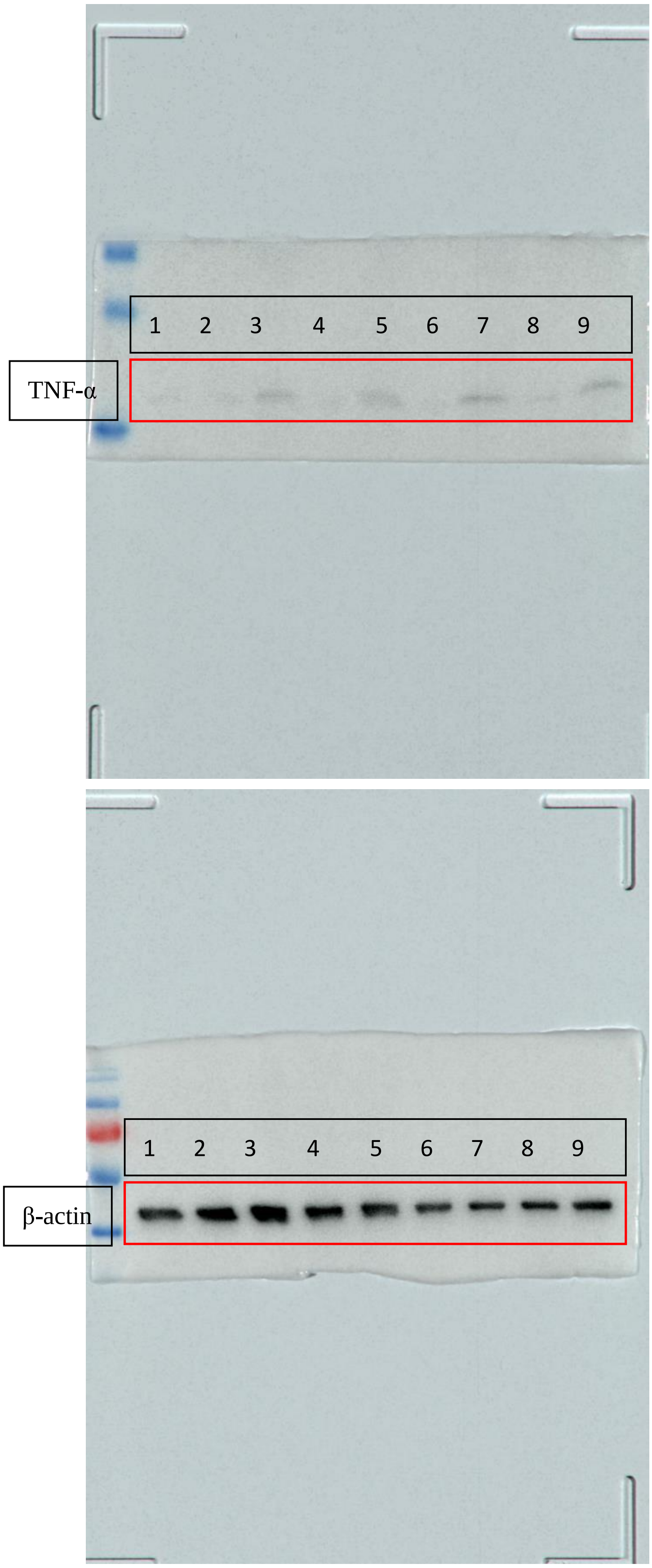

1. Control
2. 341215
3. 341215+SB203580
4. 340542
5. 340542+SB203580
6. 130803
7. 130803+SB203580
8. 360624
9. 360624+SB203580

H, J. Relative expression of TNF-α

Cell lines	Experimental group	Relative expression
HBMEC	CTR	1.01±0.08
	341215 + E2	0.88±0.29
	341215 + E2 + SB203580	3.08±0.05
	340542 + E2	1.06±0.04
	340542 + E2 + SB203580	2.59±0.10
	130803 + E2	0.97±0.07
	130803 + E2 + SB203580	2.94±0.21
	360624 + E2	1.11±0.09
	360624 + E2+ SB203580	2.04±0.18
MEC	CTR	1.00±0.02
	341215 + E2	1.12±0.38
	341215 + E2 + SB203580	1.63±0.14
	340542 + E2	0.98±0.03
	340542 + E2 + SB203580	1.38±0.20
	130803 + E2	1.00±0.09
	130803 + E2 + SB203580	1.59±0.11
	360624 + E2	0.99±0.15
	360624 + E2 + SB203580	1.40±0.09

Figure 5A-D Original gel scanning and relative expression data

A. Original gel scanning in HBMEC cells

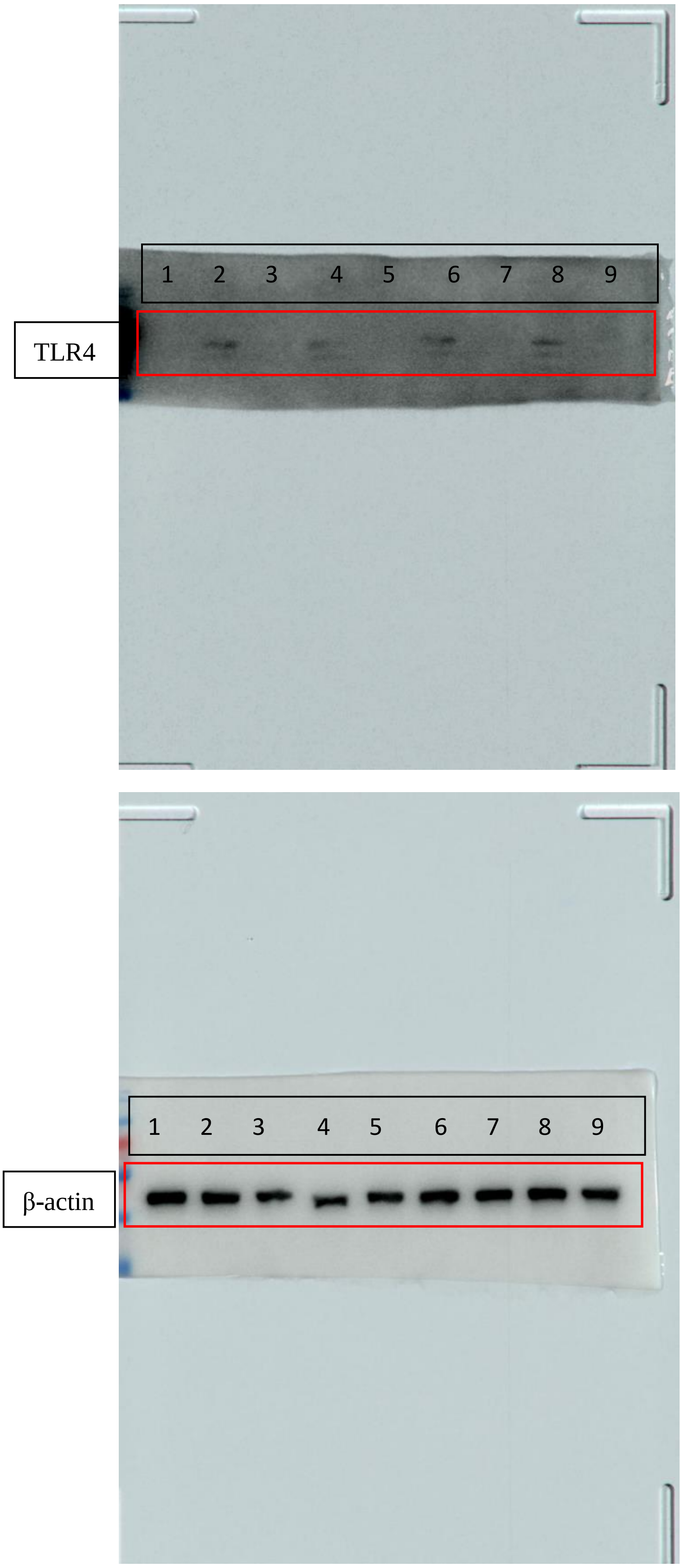

1. Negative Control
2. 341215
3. 341215+TLR4 ShRNA
4. 340542
5. 340542+TLR4 ShRNA
6. 130803
7. 130803+TLR4 ShRNA
8. 360624
9. 360624+TLR4 ShRNA

C. Original gel scanning in MEC cells

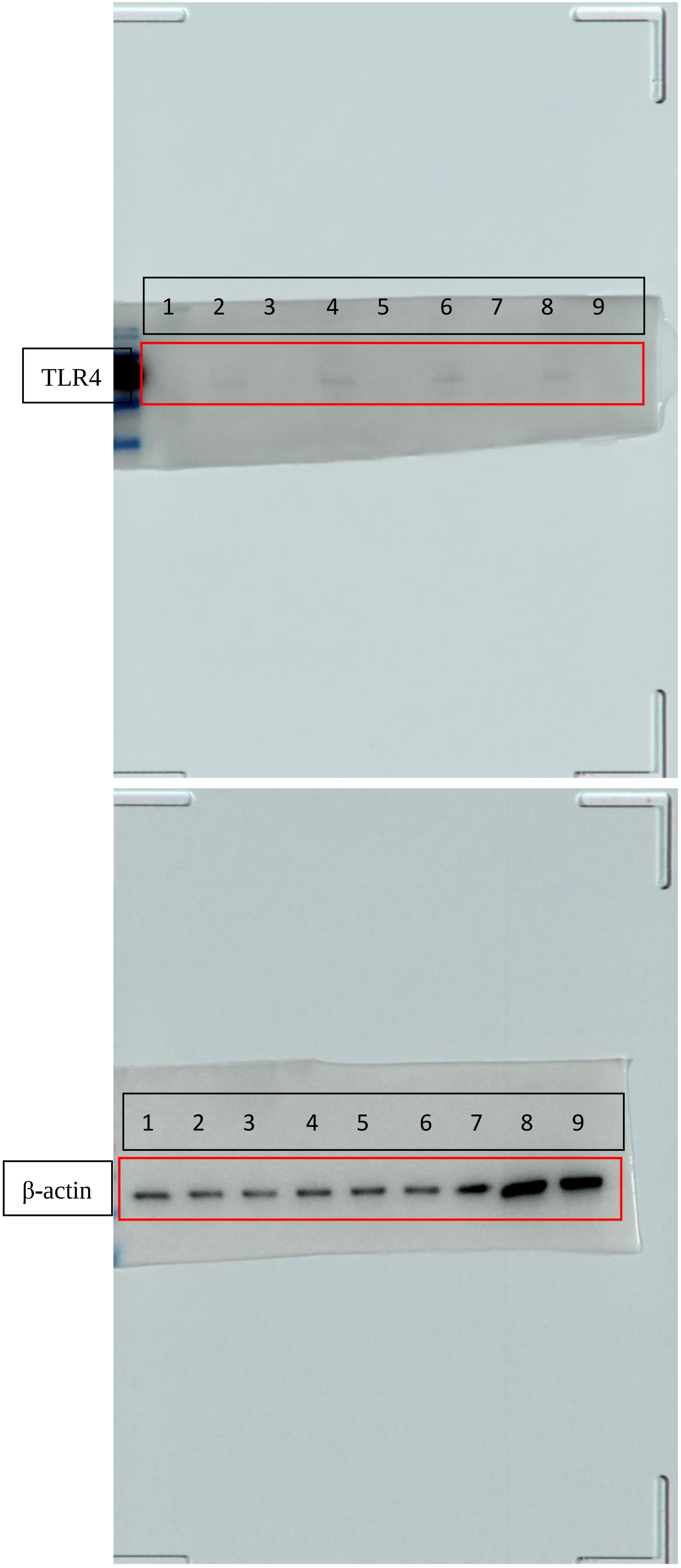

1. Negative Control
2. 341215
3. 341215+TLR4 ShRNA
4. 340542
5. 340542+TLR4 ShRNA
6. 130803
7. 130803+TLR4 ShRNA
8. 360624
9. 360624+TLR4 ShRNA

B, D. Relative expression of TLR4

Cell lines	Experimental group	Relative expression
HBMEC	Negative Control	1.00±0.21
	341215	5.13±0.29
	341215 + TLR4 shRNA	1.04±0.14
	340542	1.98±0.27
	340542 + TLR4 shRNA	1.02±0.19
	130803	3.12±0.30
	130803 + TLR4 shRNA	1.10±0.07
	360624	4.43±0.59
	360624 + TLR4 shRNA	1.14±0.00
MEC	Negative Control	1.00±0.06
	341215	1.57±0.20
	341215 + TLR4 shRNA	1.06±0.08
	340542	1.63±0.05
	340542 + TLR4 shRNA	1.00±0.01
	130803	1.70±0.15
	130803 + TLR4 shRNA	0.90±0.07
	360624	1.16±0.02
	360624 + TLR4 shRNA	0.74±0.06

Figure 5E-F. Inflammatory cytokines release in HBMEC and MEC cell lines

Cell lines	Experimental group	Inflammatory cytokines（pg/ml）		
		IL-6	IL-8	TNF-α
HBMEC	E2+341215+shRNA	368.54±21.06	503.57±63.12	46.34±2.11
	E2+340542+shRNA	341.72±26.48	516.88±22.28	45.58±2.94
	E2+130803+shRNA	137.87±3.18	281.82±11.54	30.25±3.39
	E2+360624+shRNA	125.22±19.61	207.33±29.20	22.84±4.41
MEC	E2+341215+shRNA	397.97±5.86	514.97±69.39	54.33±6.03
	E2+340542+shRNA	427.34±33.27	520.46±12.14	51.76±6.15
	E2+130803+shRNA	206.89±7.32	186.19±14.92	33.82±4.17
	E2+360624+shRNA	180.68±0.73	191.49±16.68	23.99±3.70

Figure 5G-I Original gel scanning and relative expression data

G. Original gel scanning in HBMEC cells

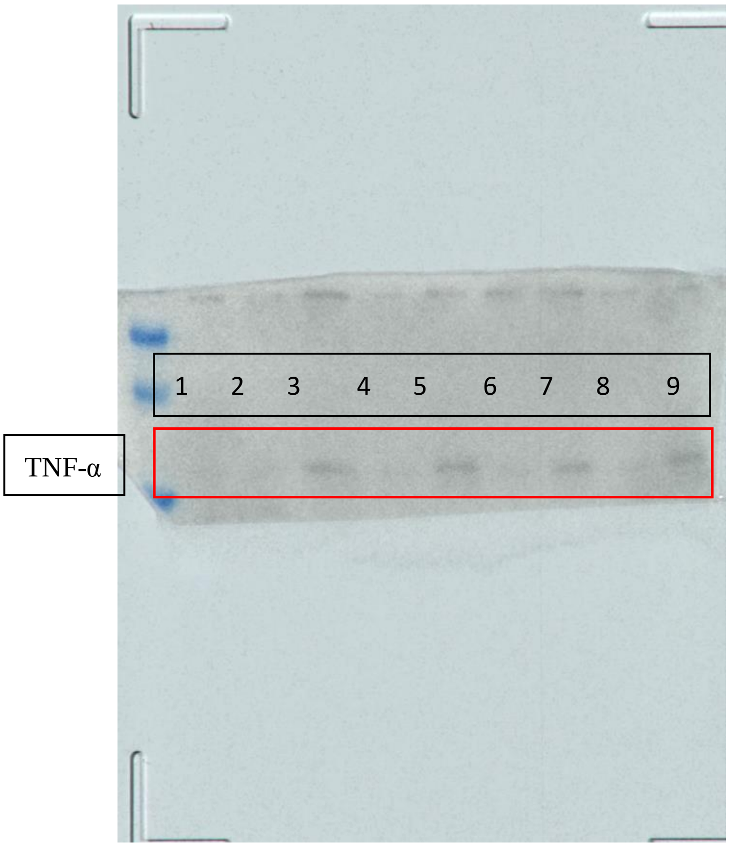

1. Negative Control
2. 341215
3. 341215+TLR4 ShRNA
4. 340542
5. 340542+TLR4 ShRNA
6. 130803
7. 130803+TLR4 ShRNA
8. 360624
9. 360624+TLR4 ShRNA

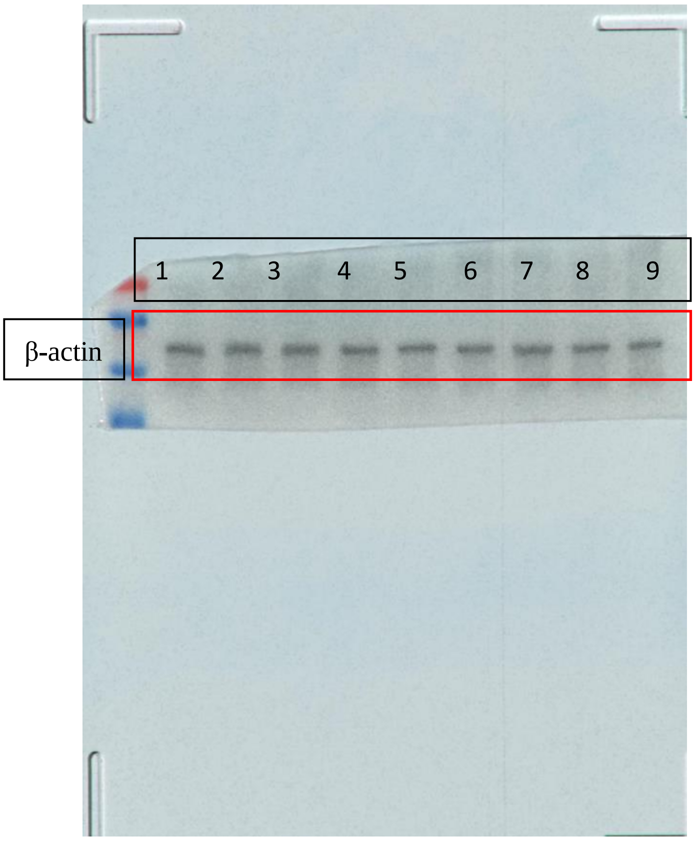

I. Original gel scanning in HBMEC cells

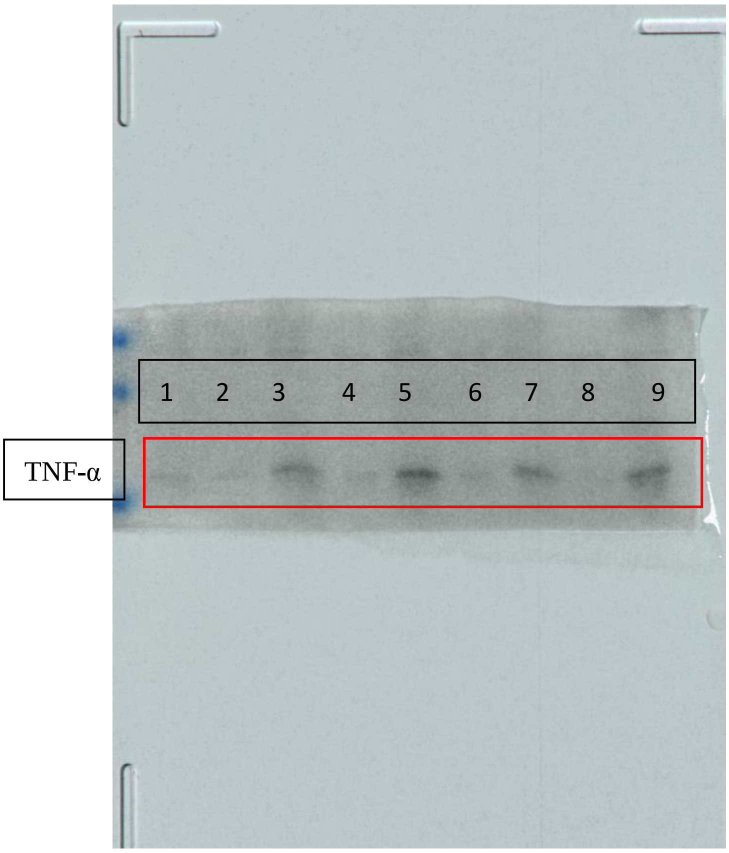

1. Negative Control
2. 341215
3. 341215+TLR4 ShRNA
4. 340542
5. 340542+TLR4 ShRNA
6. 130803
7. 130803+TLR4 ShRNA
8. 360624
9. 360624+TLR4 ShRNA

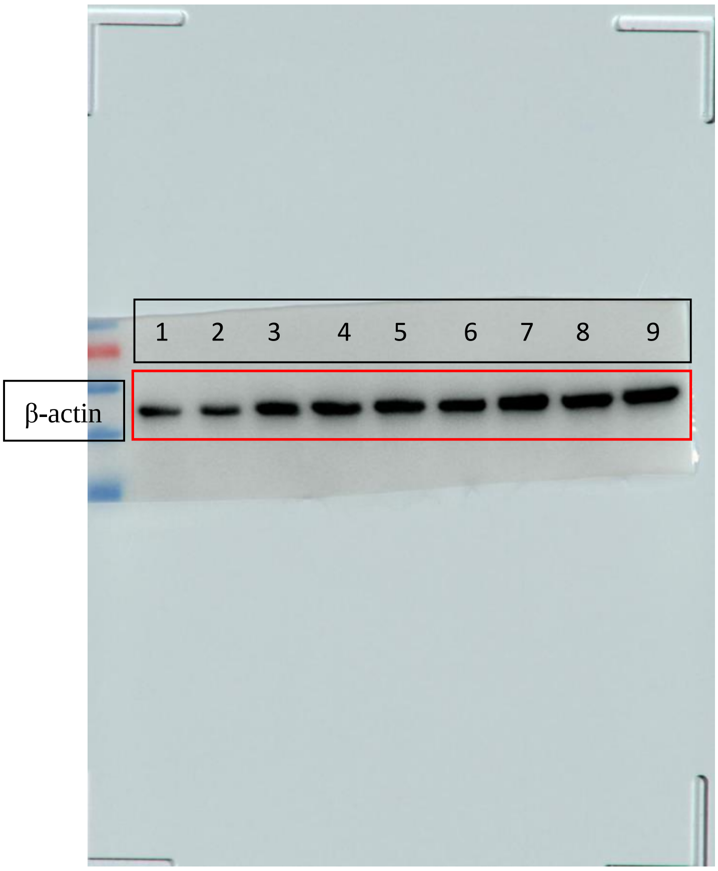

H, J. Relative expression of TNF-α

Cell lines	Experimental groups	Relative expression
HBMEC	Negative Control	1.00±0.16
	341215 + E2	1.03±0.15
	341215 + E2 + TLR4 shRNA	1.98±0.22
	340542 + E2	1.04±0.10
	340542 + E2 + TLR4 shRNA	2.13±0.26
	130803 + E2	0.96±0.08
	130803 + E2 + TLR4 shRNA	1.97±0.26
	360624 + E2	1.21±0.39
	360624 + E2+ TLR4 shRNA	2.05±0.12
MEC	Negative Control	1.02±0.17
	341215 + E2	1.20±0.26
	341215 + E2 + TLR4 shRNA	2.98±0.47
	340542 + E2	1.01±0.06
	340542 + E2 + TLR4 shRNA	5.43±0.43
	130803 + E2	0.98±0.16
	130803 + E2 + TLR4 shRNA	2.83±0.29
	360624 + E2	1.01±0.02
	360624 + E2 + TLR4 shRNA	3.13±0.14

Figure 6A-B. Inflammatory cytokines release in HBMEC and MEC cell lines

Cell lines	Experimental group	Inflammatory cytokines（pg/ml）		
		IL-6	IL-8	TNF-α
HBMEC	E2+341215+PHTPP	319.67±66.66	498.31±15.66	43.99±3.21
	E2+340542+PHTPP	340.67±56.68	535.30±51.10	53.50±10.42
	E2+130803+PHTPP	137.10±11.17	296.33±41.89	25.60±3.99
	E2+360624+PHTPP	118.93±12.41	243.17±9.60	25.78±2.50
MEC	E2+341215+PHTPP	437.38±23.35	527.41±18.04	59.73±2.19
	E2+340542+PHTPP	425.55±25.87	535.78±42.46	55.16±4.53
	E2+130803+PHTPP	228.67±33.62	172.83±18.24	30.30±1.16
	E2+360624+PHTPP	191.52±38.17	202.67±11.74	23.20±2.79

Figure 6C-D Original gel scanning and relative expression data

C Original gel scanning of TNF-α

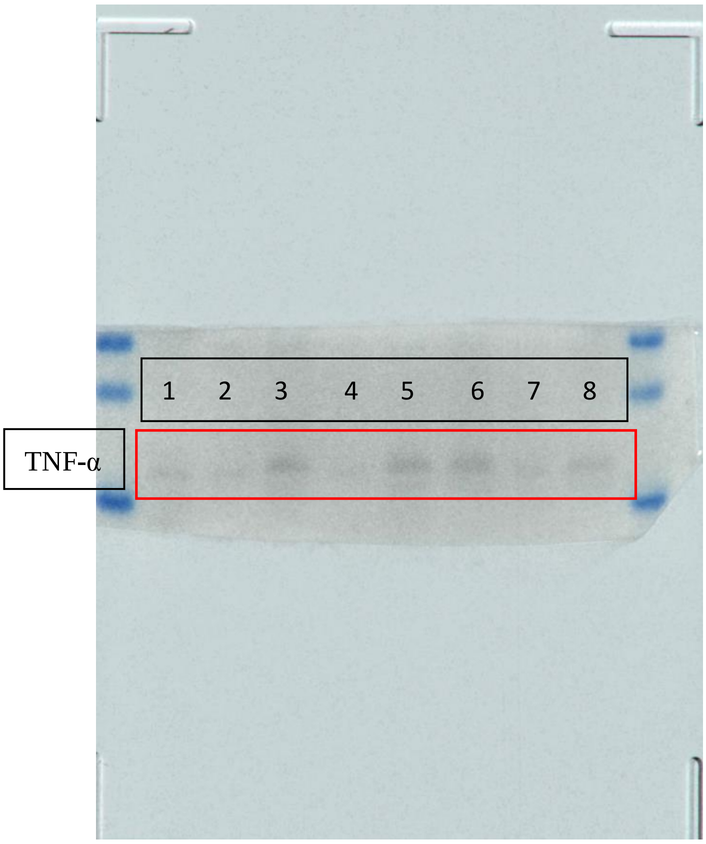

HBMEC cell line
1. Control
2. E2
3. 341215
4. E2+341215
5. E2+341215+PHTPP
6. 340542
7. E2+340542
8. E2+340542+PHTPP

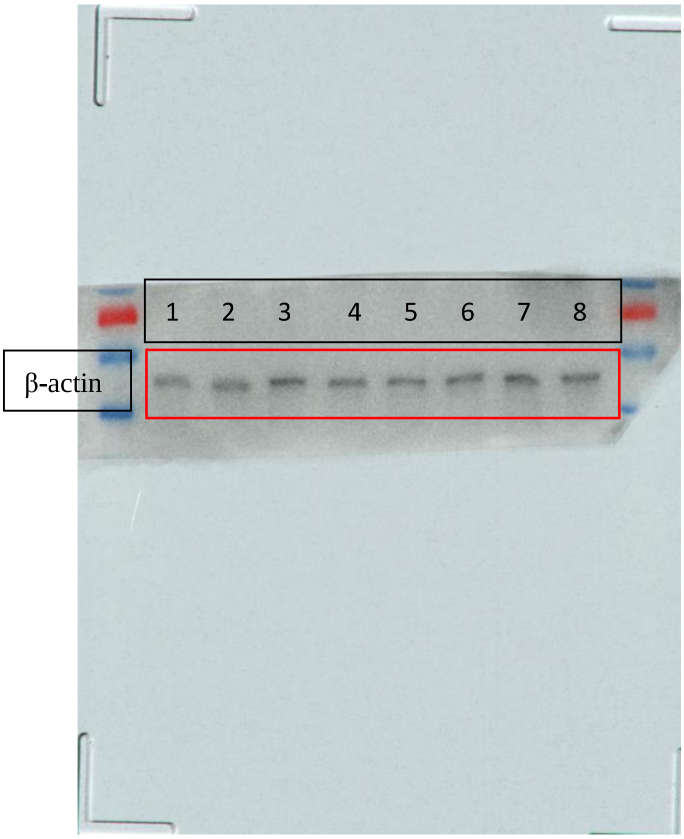

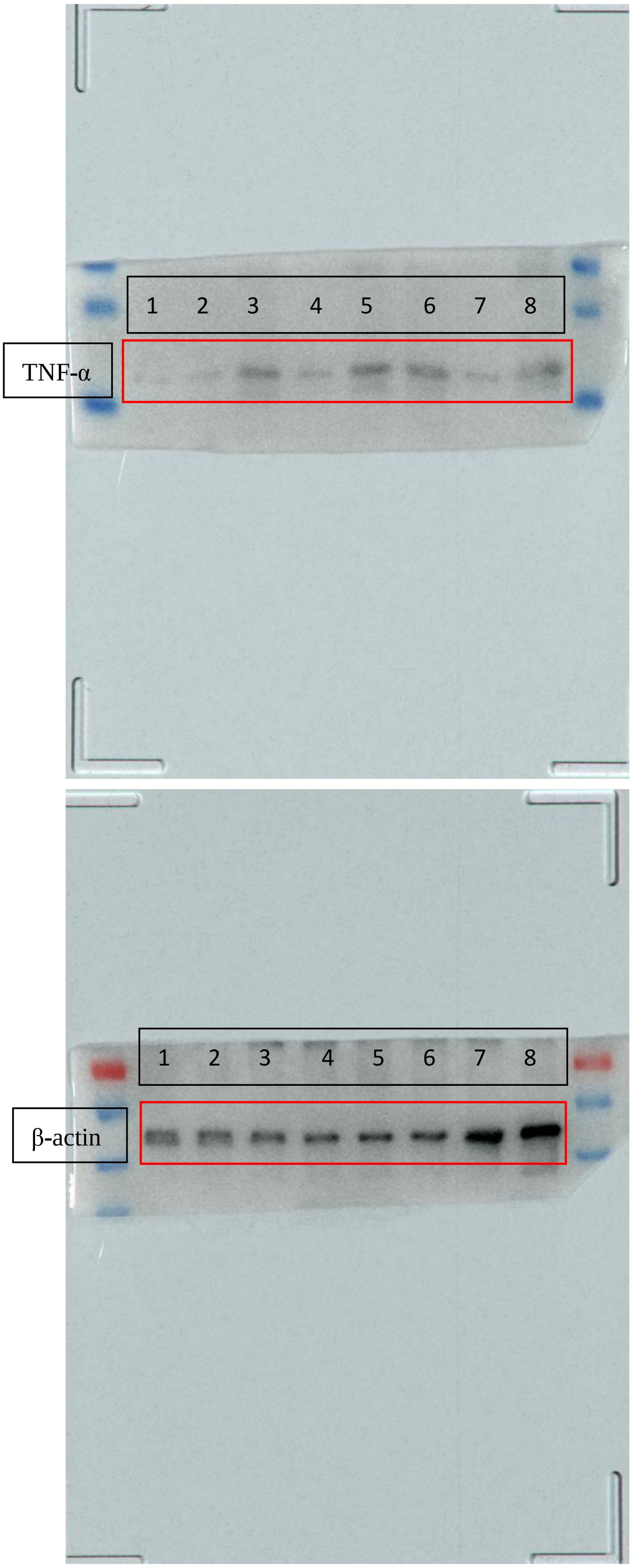

MEC cell line

1. Control
2. E2
3. 341215
4. E2+341215
5. E2+341215+PHTPP
6. 340542
7. E2+340542
8. E2+340542+PHTPP

D Original gel scanning of TNF-α

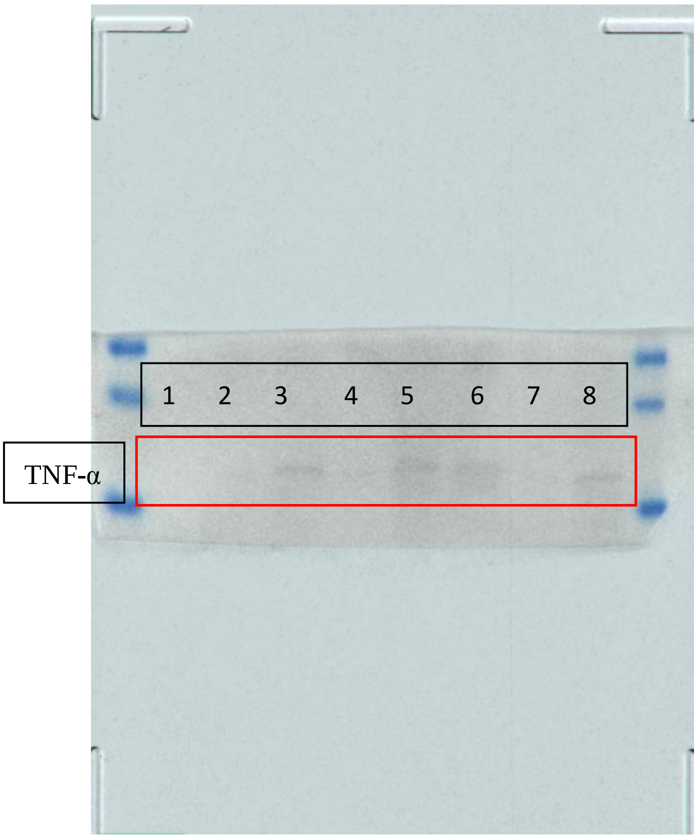

HBMEC cell line
1. Control
2. E2
3. 130803
4. E2+130803
5. E2+130803+PHTPP
6. 360624
7. E2+360624
8. E2+360624+PHTPP

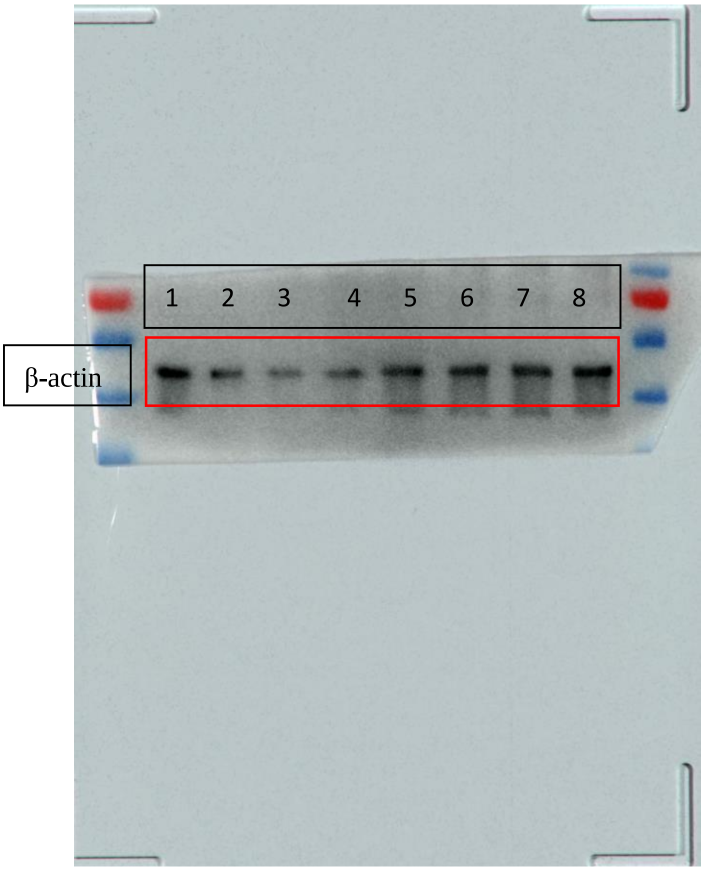

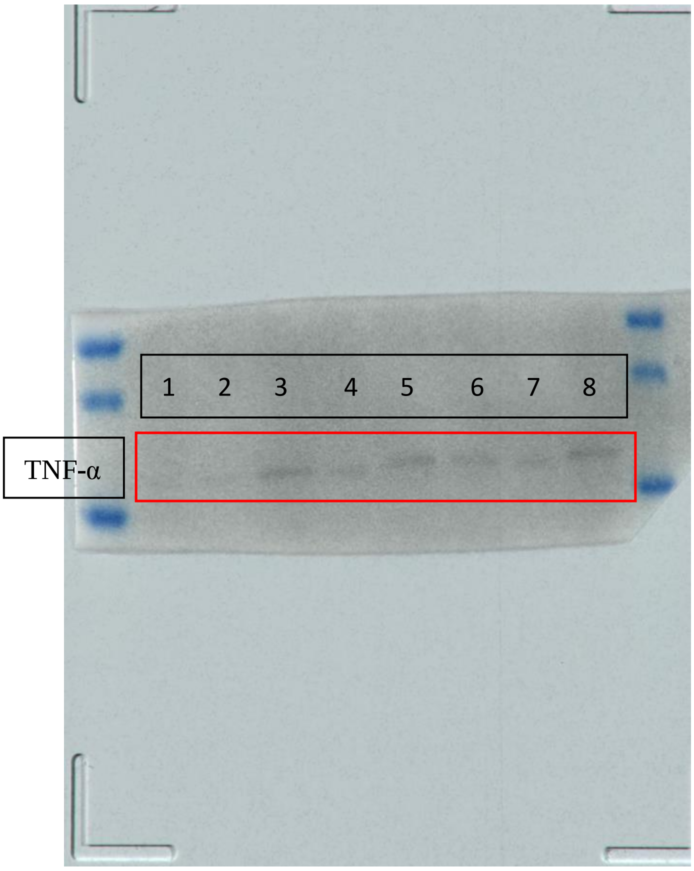

MEC cell line
1. Control
2. E2
3. 130803
4. E2+130803
5. E2+130803+PHTPP
6. 360624
7. E2+360624
8. E2+360624+PHTPP

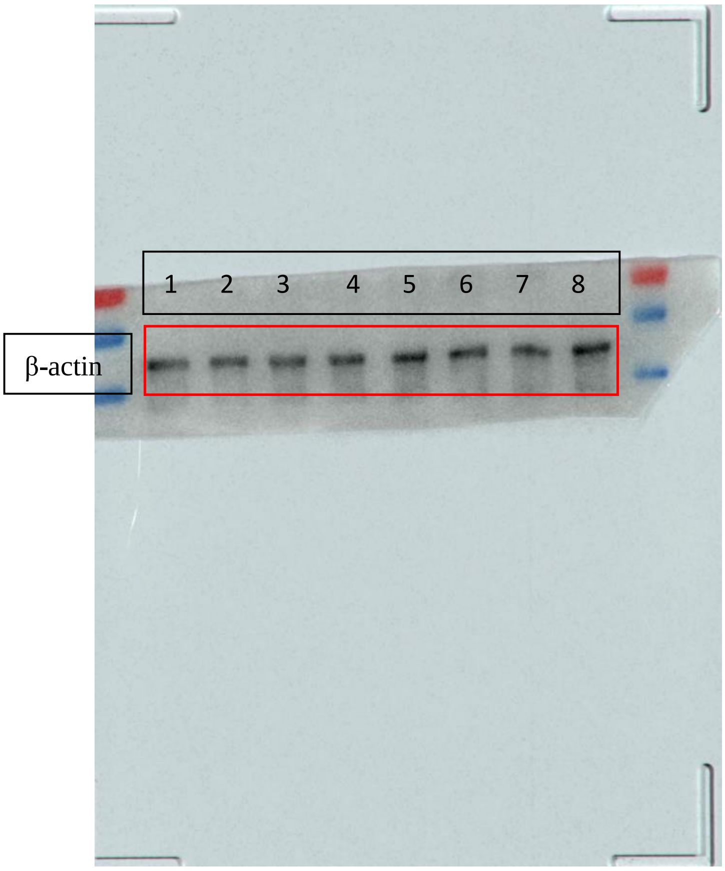

C-D Relative expression of TNF-α

Cell lines	Experimental group	Relative expression
HBMEC	CTR	1.00±0.16
	E2	0.91±0.40
	341215	2.07±0.16
	341215 + E2	0.90±0.12
	341215 + E2 + PHTPP	1.87±0.01
	340542	1.83±0.02
	340542 + E2	0.92±0.06
	340542 + E2 + PHTPP	1.60±0.05
MEC	CTR	1.01±0.11
	E2	0.96±0.14
	341215	3.01±0.28
	341215 + E2	1.31±0.12
	341215 + E2 + PHTPP	3.95±0.40
	340542	3.67±0.32
	340542 + E2	1.28±0.12
	340542 + E2 + PHTPP	2.76±0.36

Cell lines	Experimental group	Relative expression
HBMEC	CTR	1.00±0.15
HBMEC	E2	0.98±0.03
HBMEC	130803	1.53±0.13
HBMEC	130803 + E2	0.90±0.08
HBMEC	130803 + E2 + PHTPP	1.57±0.16
HBMEC	360624	1.48±0.16
HBMEC	360624 + E2	0.88±0.09
HBMEC	360624 + E2 + PHTPP	1.55±0.19
MEC	CTR	1.01±0.04
MEC	E2	1.02±0.13
MEC	130803	1.79±0.19
MEC	130803 + E2	0.99±0.03
MEC	130803 + E2 + PHTPP	1.97±0.25
MEC	360624	1.55±0.03
MEC	360624 + E2	1.35±0.12
MEC	360624 + E2 + PHTPP	2.32±0.12

Figure 6E-F Original gel scanning and relative expression data

E. Original gel scanning of p-P38

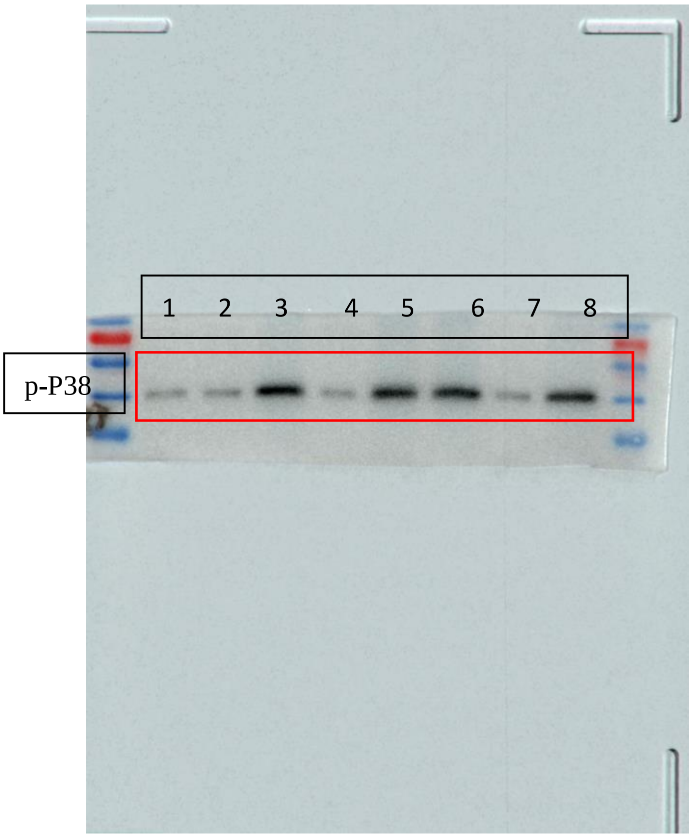

HBMEC cell line
1. Control
2. E2
3. 341215
4. E2+341215
5. E2+341215+PHTPP
6. 340542
7. E2+340542
8. E2+340542+PHTPP

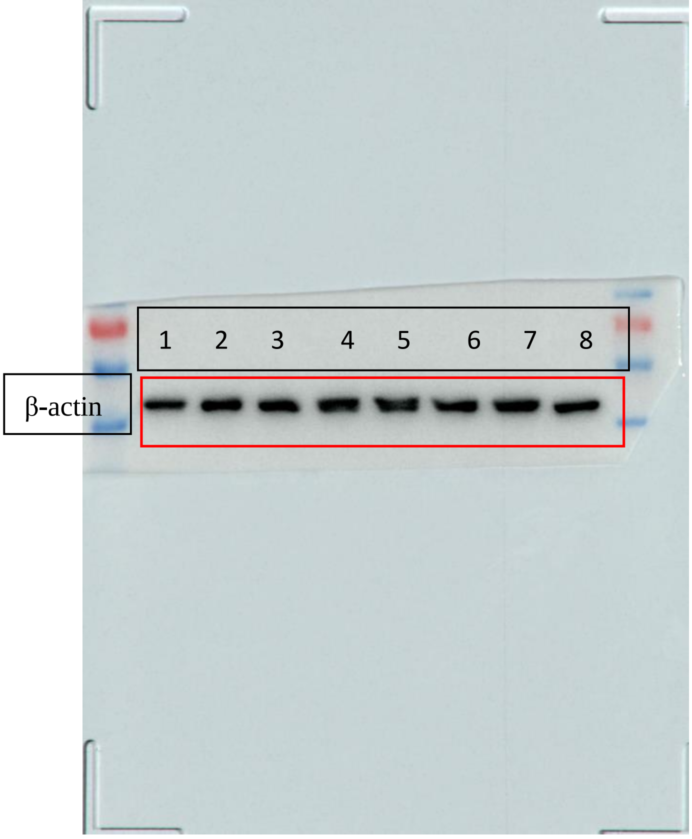

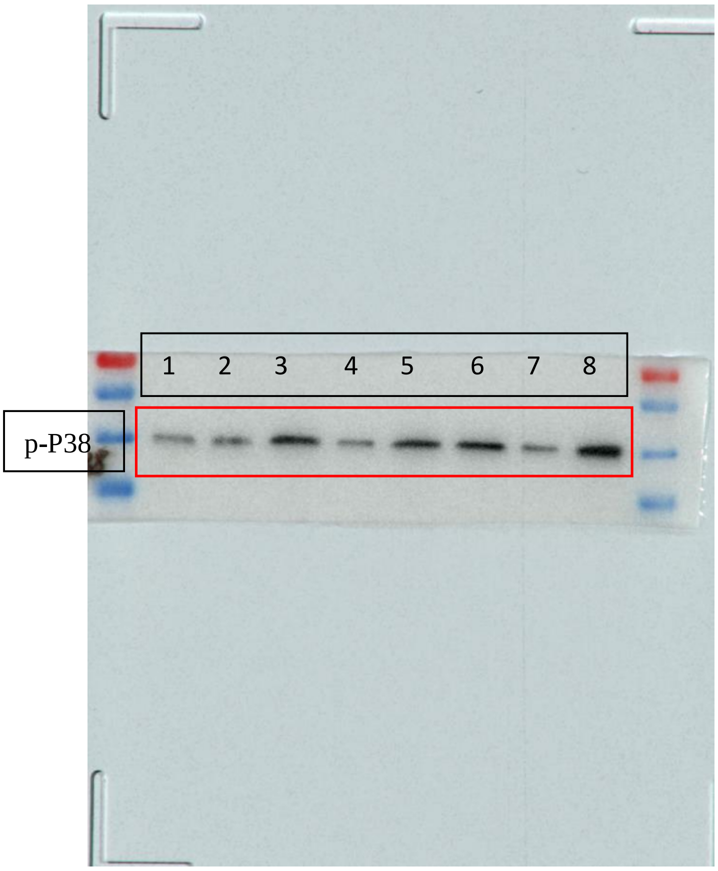

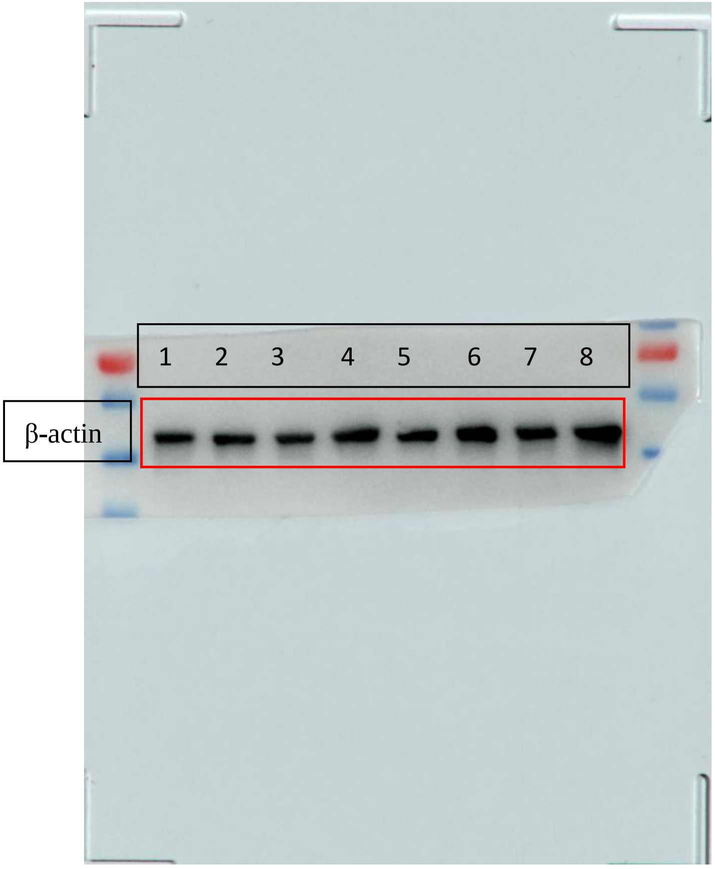

MEC cell line

1. Control
2. E2
3. 341215
4. E2+341215
5. E2+341215+PHTPP
6. 340542
7. E2+340542
8. E2+340542+PHTPP

F. Original gel scanning of p-P38

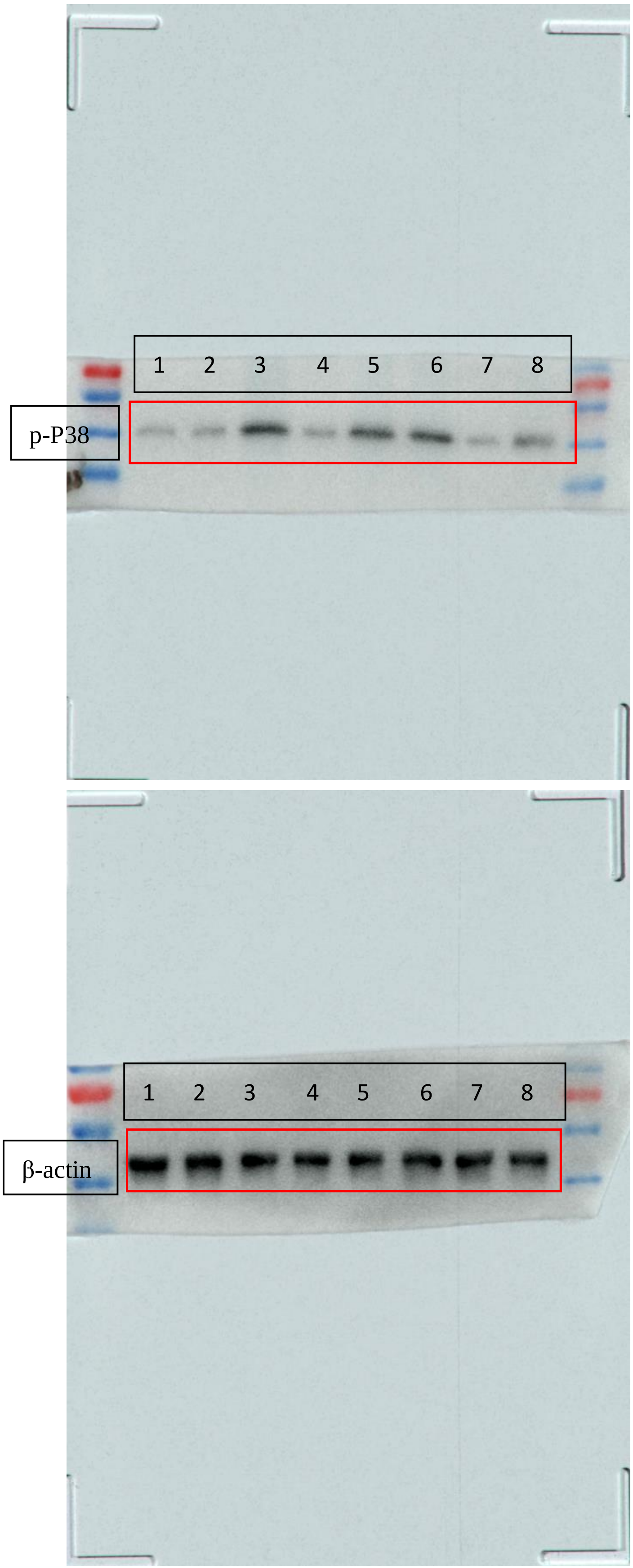

HBMEC cell line
1. Control
2. E2
3. 130803
4. E2+130803
5. E2+130803+PHTPP
6. 360624
7. E2+360624
8. E2+360624+PHTPP

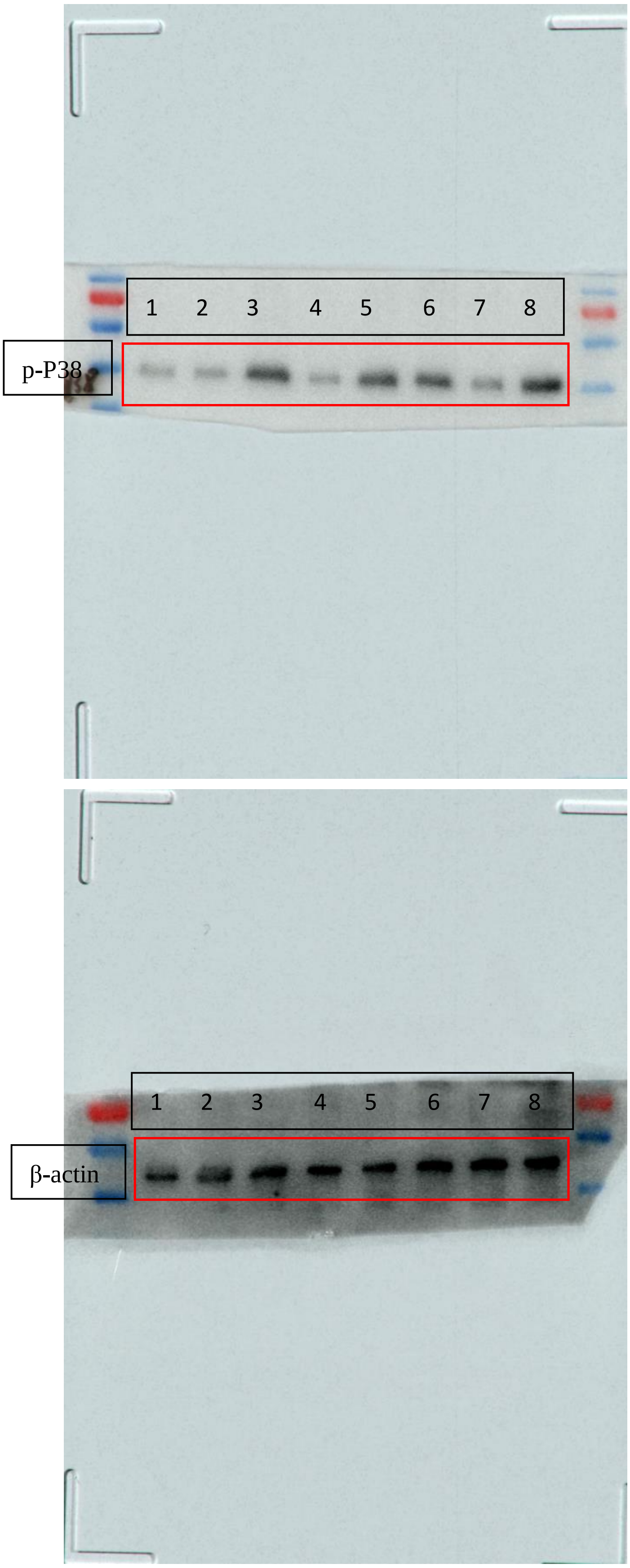

MEC cell line

1. Control
2. E2
3. 130803
4. E2+130803
5. E2+130803+PHTPP
6. 360624
7. E2+360624
8. E2+360624+PHTPP

E-F Relative expression of p-P38

Cell lines	Experimental group	Relative expression
HBMEC	CTR	1.00±0.18
	E2	1.06±0.13
	341215	6.90±0.19
	341215 + E2	1.04±0.13
	341215 + E2 + PHTPP	6.32±0.44
	340542	4.10±0.30
	340542 + E2	0.91±0.08
	340542 + E2 + PHTPP	3.81±0.53
MEC	CTR	1.01±0.01
	E2	1.00±0.00
	341215	4.24±0.33
	341215 + E2	0.92±0.08
	341215 + E2 + PHTPP	4.28±0.34
	340542	4.45±0.12
	340542 + E2	1.08±0.22
	340542 + E2 + PHTPP	5.26±0.55

Cell lines	Experimental group	Relative expression
HBMEC	CTR	1.00±0.08
	E2	0.93±0.06
	130803	3.67±0.47
	130803 + E2	0.88±0.16
	130803 + E2 + PHTPP	2.79±0.18
	360624	2.67±0.17
	360624 + E2	0.96±0.06
	360624 + E2 + PHTPP	2.04±0.25
MEC	CTR	0.98±0.04
	E2	1.04±0.26
	130803	3.48±0.10
	130803 + E2	0.71±0.04
	130803 + E2 + PHTPP	3.12±0.18
	360624	3.43±0.42
	360624 + E2	1.49±0.19
	360624 + E2 + PHTPP	3.81±0.68
