# Supplementary material for: Estrogen Suppresses Cytokines Release in cc4821 Neisseria meningitidis Infection via TLR4 and ERβ-p38-MAPK Pathway
Source: Front Microbiol. 2022 Mar 29;13:834091. doi: 10.3389/fmicb.2022.834091 (PMC9002303; doi:10.3389/fmicb.2022.834091)
Supplement: Supplementary file 2 [file Data_Sheet_2.PDF]

Supplement Table 1 Current *N. meningitidis* Isolates Information

Characteristic	Gender	
	Male	Female
Total	5707 (61.8%)	3527 (38.2%)
Dead cases	552 (58.4%)	394 (41.6%)

Supplement Table 2 Information of Isolates from Infected-male

Serogroup	Number
A	27 (17.09%)
B	34 (21.52%)
C	74 (46.84%)
E	1 (0.63%)
X	11 (6.96%)
W	11 (6.96%)
Total	158

Supplement Figures

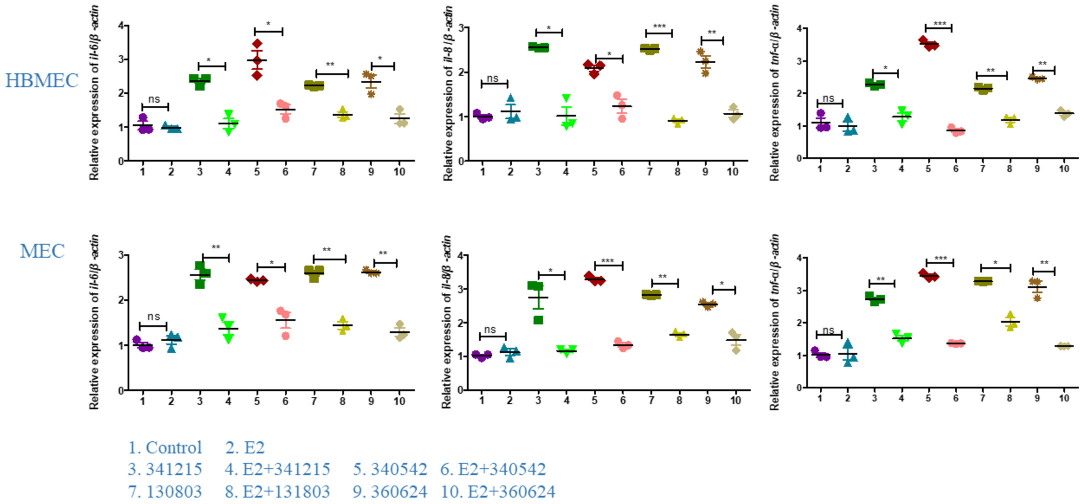

Supplement Fig.1 Expression of *il-6*, *il-8* and *tnf-α* in mRNA level

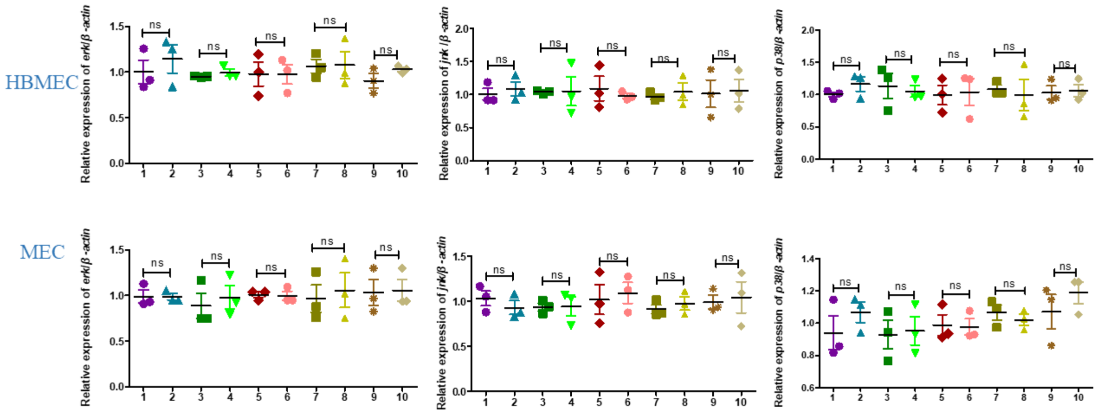

1. Control 2. E2
3. 341215 4. E2+341215 5. 340542 6. E2+340542
7. 130803 8. E2+131803 9. 360624 10. E2+360624

Supplement Fig.2 Expression of *erk*, *jnk* and *p38* in mRNA level

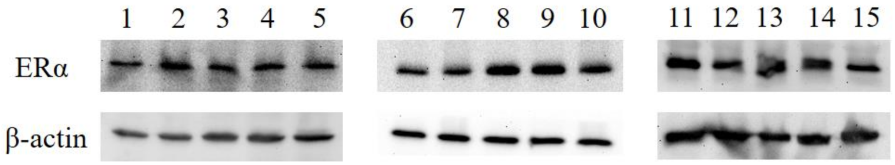

Canal 1: Control Canal 2: 341215 Canal 3: 340542 Canal 4: 130803 Canal 5: 360624

Canal 6: Control Canal 7: 440529 Canal 8: 130508 Canal 9: 310501 Canal 10: 510612

Canal 11: Control Canal 12: 150720 Canal 13: 130817 Canal 14: 340809 Canal 15: 211002

Supplement Fig.3 Expression of ERα after *N. meningitidis* infection

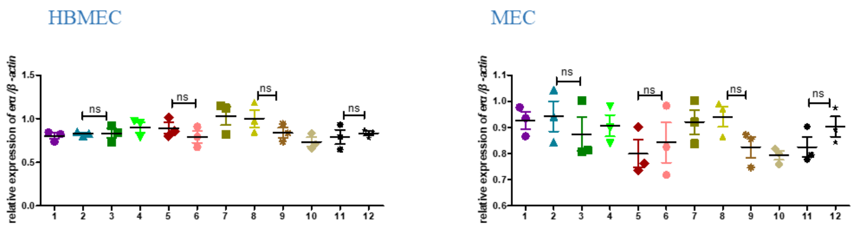

1. 341215 2. E2+341215 3. E2+341215+PHTPP
4. 340542 5. E2+340542 6. E2+340542+PHTPP
7. 130803 8. E2+130803 9. E2+130803+PHTPP
10. 360624 11. E2+360624 12. E2+360624+PHTPP

Supplement Fig.4 Relative Expression of *erα* after PHTPP Treatment
